# Sox6 Is Necessary for Efficient Erythropoiesis in Adult Mice under Physiological and Anemia-Induced Stress Conditions

**DOI:** 10.1371/journal.pone.0012088

**Published:** 2010-08-09

**Authors:** Bogdan Dumitriu, Pallavi Bhattaram, Peter Dy, Yuanshuai Huang, Nayeem Quayum, Jan Jensen, Véronique Lefebvre

**Affiliations:** 1 Department of Cell Biology and Orthopaedic Research Center, Cleveland Clinic Lerner Research Institute, Cleveland, Ohio, United States of America; 2 Department of Internal Medicine, Cleveland Clinic Lerner Research Institute, Cleveland, Ohio, United States of America; 3 Department of Stem Cell Biology and Regenerative Medicine, Cleveland Clinic Lerner Research Institute, Cleveland, Ohio, United States of America; National Institute on Aging, National Institutes of Health, United States of America

## Abstract

**Background:**

Definitive erythropoiesis is a vital process throughout life. Both its basal activity under physiological conditions and its increased activity under anemia-induced stress conditions are highly stimulated by the hormone erythropoietin. The transcription factor Sox6 was previously shown to enhance fetal erythropoiesis together and beyond erythropoietin signaling, but its importance in adulthood and mechanisms of action remain unknown. We used here *Sox6* conditional null mice and molecular assays to address these questions.

**Methodology/Principal Findings:**

*Sox6^fl/fl^ErGFPCre* adult mice, which lacked *Sox6* in erythroid cells, exhibited compensated anemia, erythroid cell developmental defects, and anisocytotic, short-lived red cells under physiological conditions, proving that Sox6 promotes basal erythropoiesis. Tamoxifen treatment of *Sox6^fl/fl^CaggCreER* mice induced widespread inactivation of *Sox6* in a timely controlled manner and resulted in erythroblast defects before reticulocytosis, demonstrating that impaired erythropoiesis is a primary cause rather than consequence of anemia in the absence of *Sox6*. Twenty five percent of *Sox6^fl/fl^ErGFPCre* mice died 4 or 5 days after induction of acute anemia with phenylhydrazine. The others recovered slowly. They promptly increased their erythropoietin level and amplified their erythroid progenitor pool, but then exhibited severe erythroblast and reticulocyte defects. Sox6 is thus essential in the maturation phase of stress erythropoiesis that follows the erythropoietin-dependent amplification phase. *Sox6* inactivation resulted in upregulation of embryonic globin genes, but embryonic globin chains remained scarce and apparently inconsequential. *Sox6* inactivation also resulted in downregulation of erythroid terminal markers, including the *Bcl2l1* gene for the anti-apoptotic factor Bcl-xL, and in vitro assays indicated that Sox6 directly upregulates *Bcl2l1* downstream of and beyond erythropoietin signaling.

**Conclusions/Significance:**

This study demonstrates that Sox6 is necessary for efficient erythropoiesis in adult mice under both basal and stress conditions. It is primarily involved in enhancing the survival rate and maturation process of erythroid cells and acts at least in part by upregulating *Bcl2l1*.

## Introduction

Erythropoiesis or red blood cell formation is a vital process throughout life. It first occurs in the yolk sac of the mammalian embryo. Then referred to as embryonic or primitive erythropoiesis, it produces red blood cells (RBCs) that are large, nucleated, and short-lived, and that contain hemoglobin made with the products of embryonic globin genes. Embryonic erythropoiesis is taken over by definitive erythropoiesis when the liver starts developing around day 10 of mouse gestation. This organ is the fetus erythropoietic center. It releases RBCs that are small, enucleated, long-lived, and whose hemoglobin is made with the products of the alpha and beta adult globin genes. Erythropoiesis transiently switches to the spleen around birth, before primarily and definitively homing to the bone marrow. It remains a very active process throughout postnatal development, constantly adjusting its RBC output to the growing body size. Under physiological conditions, a healthy adult mouse or human maintains a basal level of erythropoietic activity in the bone marrow, renewing 1–2% of its RBC population on a daily basis. A number of conditions that result in severe hypoxia force erythropoiesis to quickly and efficiently increase its output and eventually renew the entire pool of RBCs in just a few days. This accelerated process is called stress erythropoiesis and mainly occurs in the spleen [1]. In the most severe cases, it is triggered by acute anemia due to hematological disorders, severe blood loss, or chemotherapy. These conditions can be fatal if stress erythropoiesis is impaired. It is therefore important to fully understand the molecular mechanisms that underlie basal and stress erythropoiesis to be able to propose improved strategies to stimulate stress erythropoiesis in patients with such conditions.

Basal and stress erythropoiesis occur in several developmental cell stages and are controlled by complex molecular networks. Specific transcription factors govern the recruitment of hematopoietic stem cells to the erythroid lineage and the sequential differentiation and maturation of the cells into erythroid progenitors, proerythroblasts, erythroblasts, reticulocytes, and erythrocytes [2]–[4]. Best known are the Gata1 and Fog1 zinc finger factors, the Eklf erythroid Krüppel-like factor, and the Foxo3a forkhead factor [5]–[10]. Erythroid cell survival and proliferation are largely controlled by the hormone erythropoietin (Epo) [11]–[13], but also by the stem cell factor, glucocorticoids, and bone morphogenetic protein-4 [14]–[16]. Epo is produced by the kidney at a low level under basal conditions, and at a very high level in response to hypoxia. It binds to a specific receptor, EpoR, expressed almost exclusively on erythroid cells, and signals principally through the Jak/Stat and Akt/PI3K pathways. Many factors necessary for basal erythropoiesis, such as Epo and Gata1, are also required for stress erythropoiesis [1], [17], [18]. In contrast, molecules like the Stat5 transcription factor and Bcl-xL anti-apoptotic factor are dispensable for basal erythropoiesis, but essential to ensure the high efficiency of stress erythropoiesis downstream of Epo [19]–[22].

Sox6 is a transcription factor with an Sry-related high-mobility-group DNA-binding (Sox) domain and a highly conserved coiled-coil homodimerization domain, but with no known transactivation or transrepression domain [23]. While many Sox proteins are required for cell fate specification and differentiation in discrete lineages, Sox6 forms with Sox5 and Sox13 a Sox group (SoxD) that act as significant modulators rather than obligatory determinants of cell fate and differentiation. For instance, Sox6 is co-expressed with Sox5 in chondrocytes and the two proteins act redundantly to potentiate transactivation of cartilage marker genes by the chondrogenic master Sox9 and thereby to achieve efficient chondrogenesis [24], [25]. Sox5 and Sox6 are also co-expressed and acting redundantly in oligodendrocytes [26]. Their roles in this lineage are to delay gliogenesis by blocking the ability of Sox9 and Sox10 to bind and transactivate oligodendrocyte markers. Sox6 and its relatives thus have the ability to enhance as well as repress gene transcription. The determinants of their positive and negative influence on transcription include specific gene sequences and protein partners [23]. *Sox6^−/−^* mice die at birth or in the third week of age with skeletal, glial, and cardiac developmental defects [24]–[29]. They also feature a large proportion of nucleated definitive red blood cells and are anemic, despite an increased Epo level and liver volume [30]. Sox6, but neither Sox5 nor Sox13, is expressed in definitive erythroid cells and has cell-autonomous roles in these cells [30], [31]. It potentiates the ability of Epo to stimulate early erythroid cell survival and proliferation, and it also facilitates erythroblast and reticulocyte maturation, but the molecular mechanisms underlying these functions remain unknown [30]. The embryonic globin genes are currently the only genes whose expression is known to be altered in *Sox6^−/−^* erythroid cells. While these genes are silent in normal definitive erythroid cells, they are highly expressed in *Sox6^−/−^* cells. Sox6 was shown to bind to the proximal promoter of the embryonic epsilon-globin gene and to interact with Bcl11a to repress epsilon-globin gene expression [31], [32]. It is unknown, however, whether ectopic expression of embryonic globins is responsible for the survival, proliferation or maturation defects of *Sox6^−/−^* erythroid cells or whether Sox6 also modulates the expression of other genes in erythroid cells. *Sox6* ^−/−^/wild-type hematopoietic chimeras were shown to maintain expression of the embryonic epsilon-globin gene in adulthood and to have misshapen RBCs [33], but these mice did not address the question of the need for Sox6 in basal or stress erythropoiesis in adulthood.

In light of the importance of basal and stress erythropoiesis in adulthood, we undertook the present study to further uncover the importance and molecular roles of Sox6 in this process. We show that Sox6 continues to enhance erythroid cell development throughout adulthood in the mouse. Its contribution is significant in basal erythropoiesis and critical to ensure mouse survival and prompt recovery in stress erythropoiesis. Embryonic globins are scarce in the developing erythroid cells and RBCs of *Sox6* mutants and their presence appears inconsequential. Sox6 effectively upregulates the expression of multiple late erythroid cell markers, and one of its direct targets is *Bcl2l1*, which encodes Bcl-xL, an important survival factor acting downstream and beyond Epo signaling in erythroid cells.

## Materials and Methods

### Mouse generation and analysis

Mice were used according to federal guidelines and as approved by the Cleveland Clinic Institutional Animal Care and Use Committee (protocol ARC 8511, originally approved on 11.29.2007 - latest annual renewal approved 11.24.2009). They were carrying *Sox6* null alleles [25], *Sox6* conditional null alleles [34], an *ErGFPCre* transgene [35], or a *CaggCreER* transgene [36], as described. Tamoxifen (Sigma-Aldrich, St Louis, MO) was dissolved at 20 mg/ml in corn oil and injected intraperitoneally at 9 mg per 40 g mouse 3 times at 2-day intervals [36]. Phenylhydrazine (Sigma) was dissolved at 6 mg/ml in PBS and injected intraperitoneally at 60 mg/kg on two consecutive days [37]. All mice were on a mixed 129× C57BL/6J genetic background. Complete blood cell tests, serum Epo level assays, fluorescence-activated cell sorting (FACS) analyses, and globin chain and ghost analyses were performed as described [30]. CD44 antibody was purchased from BD Biosciences (San Jose, CA). The life span of RBCs was measured by injecting 3 mg EZ-Link-sulfo-NHS-Biotin (Pierce, Rockford, IL) in adult mice and measuring the decay of labeled RBCs by fluorescence-activated cell sorting (FACS) using streptavidin-phycoerythrin [38]. FACS was carried out using an LSRII instrument (BD Biosciences) and data were analyzed using FlowJo software (Tree Star, Ashland, OR). For the assay of reactive oxygen species (ROS), blood cells were suspended in PBS supplemented with 2% FCS and loaded with 5 µM 5-(and 6-)-chloromethyl-2′,7′-dichlorodihydrofluorescein diacetate (CM-H2DCFDA; Invitrogen, Carlsbad, CA) in the dark for 20 minutes at 37°C, 5% CO_2_. The oxidative conversion of CM-H2DCFDA to its fluorescent product was measured immediately by flow cytometry. A positive control was created by incubating cells with 3 mM tert-butylhydroperoxide (Invitrogen). Western blot with a hemoglobin beta/gamma/delta/epsilon antibody (SC-22718, Santa-Cruz Biotechnology, Santa Cruz, CA) was performed using a standard protocol.

### RNA assays

Northern blot was performed with total RNA as described [30]. Probes were generated by RT-PCR and cloned in pCR4-TOPO (Invitrogen). Globin probes were generated using primers as described [31]. A 559-bp *Bcl2l1* probe was made using the forward primer (FP) TGGTCGACTTTCTCTCCTAC and reverse primer (RP) AGAGATCCACAAAAGTGTCC. A 592-bp *Rnf11* probe was made using ACTTCGGATGACATCTCCCTGCTT (FP) and TGGCTGCAGATGTTGAGGGAAGTA (RP); a 620-bp *Tmcc2* probe was made using TGCTGAGCCTAGAGAGTGCAGAAA (FP) and ATCGAACACAACACTCCAGAGCCT (RP); and a 678-bp *Xpo7* probe was made using ATGCTGAGAGGGCCAAGTTTCTCT (FP) and GCTCACCATCCATTGCATCTTGCT (RP). Gene expression microarray screening and real-time RT-PCR were performed essentially as described [39]. Briefly, complementary RNA was hybridized to Illumina MouseRef-8 v2 expression bead chips (Illumina, San Diego, CA). Genes differentially expressed by at least 1.5 fold in 3 independent experiments were identified and analyzed in volcano plots using GeneSpeed Beta Cell software [40]. Venn diagrams were generated using BioVenn software [41]. For real-time RT-PCR, cDNA was amplified with gene-specific primers using SYBR green PCR master mix on an ABI PRISM 7900HT qPCR instrument (Applied Biosystems, Foster City, CA). Relative mRNA levels were calculated using the 2^−DD*C*T^ method. FP and RP primers were, respectively, as follows: *Bcl2l1* GCTGCATTGTTCCCGTAGAG and GTTGGATGGCCACCTATCTG; *Cd59a* GCCTCACATGCTACCACTG and CCAACACCTTTGATACACTTGC; *Cd59b* FPTTCTGGCTGTCTTCTGTTCC and TTGATACACTTGCCTTCCGG; *Eif5* CGTGTCGACCAGTTCTATCG and CTTGATTCCATTTCCTTTGCCC; *Lmo2* CATCGAAAGGAAGAGCCTGG and GTACTGGTCGATGGCTTTCAG; *Rnf11* GGAGTTTATGACCCTGGAAGAG and CAGTCCAGGTGATAGATGTGC; *St5* CCAGGGAAAACCATCAAAGTG and AGATTCGGATAAGCTGACGC; *Tmcc2* GAACTGCGGGAGATAAAGGAG and TGTTCCTCCAGCCTCTCATA; and *Xpo7* CCTATATCACATCCCGCTTGG and TTCTCATACTCACAACGCCC.

### In vitro cultures and assays

Erythroid colony formation assays and primary erythroid cell cultures were performed as described using the whole cell populations of adult erythropoietic tissues and TER119^−^ fetal liver cells, respectively [30], [42]. Reporter assays performed as described [39]. Briefly, Cos-1 cells were transfected with FuGENE6 (Roche, Madison, WI), a mouse *Bcl2l1* reporter, a Sox6 [24], constitutively active Stat5 [21] or empty expression plasmid, and a pSV2betaGal plasmid as control for transfection efficiency. The *Bcl2l1* reporter was made by cloning a 2.8-kb *Bcl2l1* gene fragment containing the upstream enhancer [43], promoter, first exon and intron, and second exon up to the first codon into the pGL3 basic reporter plasmid (Promega, Madison, WI). This fragment was amplified from mouse genomic DNA using AAGATCAAGGGCAGCCCAC (FP) and CCATGGTCCAAAACACCTGCTCACTTACTG (RP). Chromatin immunoprecipitation was performed as described [39]. Briefly, 3×10^6^ cells were crosslinked with 0.5% formaldehyde for 10 min followed by addition of glycine at 125 mM. Chromatin was sheared by sonication to fragments averaging 400 bp in buffer containing 1% SDS, 10 mM EDTA, 50 mM Tris-HCl, pH 8.1 and protease inhibitor cocktail (Sigma-Aldrich). Chromatin was pre-cleared on protein G-coupled Dynal magnetic beads (Invitrogen) followed by immunoprecipitation with antibodies against Sox6 [24] or Stat5 (sc-836X, Santa-Cruz Biotechnology) coupled to protein G-coupled Dynal magnetic beads. No antibody and non-immune IgG were used as controls. TGGAAGTCCCTTTAGGGTTTCGGA (FP) and TGGTTCCTCCATCGACCAGATCAA (RP) amplified a 236-bp *Bcl2l1* promoter region; TGTTGGACACCGACATCGAAAGGA (FP) and AGAAAGGGACTGGCATCGAGACAT (RP) amplified a 177-bp *Bcl2l1* first-intron region; and TGAGTCCTATCCTGGGAACCATCA (FP) and ATTTATAGGAACCCGGATGGTGGG (RP) amplified a 284-bp *Gapdh* promoter region. PCR was done with 1 cycle at 94°C for 3 min; 40 cycles at 94°C for 20 sec, 59°C for 30 sec, and 72°C for 30 sec; and 1 cycle at 72°C for 10 min.

### Statistical analysis

Numerical values were expressed as means ± SD. The significance of differences between control and mutant mice was determined using the statistical Student's t-test. A *p* value less than 0.05 was considered significant.

## Results

### *Sox6* is needed to facilitate erythroid development and RBC survival in basal erythropoiesis

**Figure 1:**
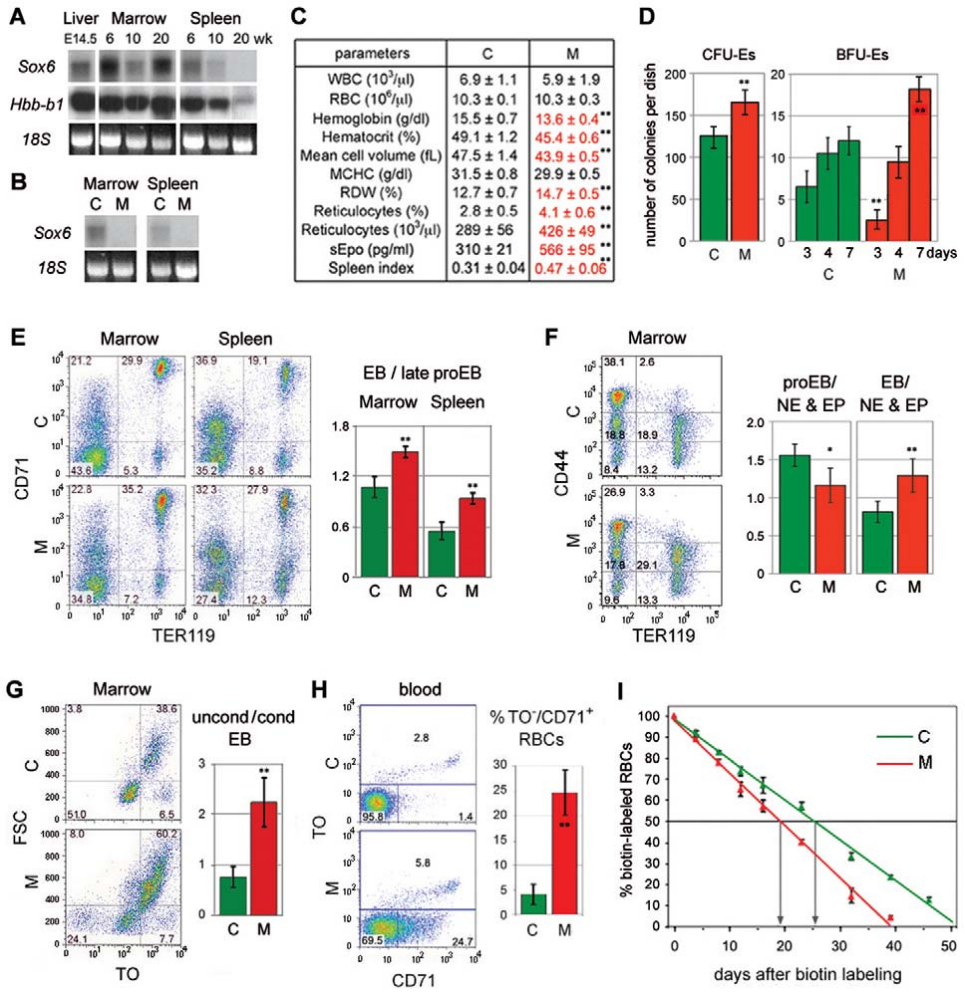
*Sox6* is expressed and needed in erythropoietic tissues of adult mice under physiological conditions. (**A**) Northern blot of RNA from liver at embryonic day 14.5 (E14.5), and marrow and spleen at 6, 10, and 20 weeks. RNA was detected with *Sox6* coding-exon-2 and beta-globin (*Hbb-b1*) probes. 18S rRNA stained with ethidium bromide is shown as loading control. The decrease in *Sox6* and *Hbb-b1* RNA level in 10-week marrow probably reflects sample variation. (**B**) Northern blot of RNA from marrow and spleen of 10-week-old *Sox6^fl/fl^* (C) and *Sox6^fl/fl^ErGFPCre* (M) mice. The *Sox6* probe was the coding exon 2, which is deleted in mutants. (**C**) Blood parameters and spleen index (percentage of spleen/body weight) in similar mice as in B. Averages with standard deviation are shown for 6 mice. Red, abnormal parameters. WBC, white blood cells; MCHC, mean cell hemoglobin concentration; RDW, red cell distribution width. (**D**) Erythroid colony formation assay using *Sox6^fl/fl^* (C) and *Sox6^fl/fl^ErGFPCre* (M) adult spleen. CFU-Es were counted after 3 days and mature BFU-Es after 3, 4, and 7 days. Columns, average with standard deviation obtained for 3 mice. **p<0.01. (**E**) FACS using CD71 and TER119. Left, representative profiles. CD71^−^/TER119^−^, non- and early erythroid cells; CD71^+^/TER119^−^, late proerythroblasts; CD71^+^/TER119^+^, erythroblasts; CD71^−^/TER119^+^, RBCs. The percentage of cells in each population is indicated. Right, ratios of erythroblasts (EB)/late proerythroblasts. The data in panels (E–H) are averages with standard deviation for 3 mice. **p<0.01. (**F**) FACS using CD44 and TER119. Left, representative profiles. Right, ratios of proerythroblasts (CD44^high^/TER119^−^)/non-erythroid (NE) and erythroid precursors (EP; CD44^−/med^/TER119^−^); and erythroblasts (CD44^high^/TER119^−^)/non-erythroid and erythroid precursors. *p<0.05, **p<0.01. (**G**) FACS of erythroblasts (TER119^+^/CD71^+^). Left, representative TO/FSC profiles. Right, ratios of FSC^high^/TO^high^ uncondensed versus FSC^low^/TO^low^ condensed erythroblasts. **p<0.01. (**H**) FACS of blood. Left, representative TO/CD71 profiles. Right, percentages of atypical reticulocytes (CD71^+^/TO^−^). **p<0.01. (**I**) Decay profiles of biotin-labeled RBCs. Each value is average with standard deviation for 6 mice. Arrows, RBC half-lives.

*Sox6* was previously shown to be expressed in definitive erythroid cells in mouse fetuses and one-week-old pups, but was not tested in adult mice [30]. Using total RNA from bone marrow and spleen of 6-, 10-, and 20-week-old mice, we found that *Sox6* remained highly expressed in bone marrow throughout adulthood (Fig. 1A). It was also highly expressed in the spleen of young adults, but became downregulated as mice aged. Expression of the adult beta-globin gene similarly decreased with age, reflecting fading of erythropoietic activity in this organ under steady-state conditions for erythropoiesis.

To test whether *Sox6* controls erythropoiesis in adulthood, we generated mice harboring *Sox6* conditional null alleles (*Sox6^fl/fl^*) [34] and the *ErGFPCre* allele [35]. *ErGFPCre* is an *EpoR* knock-in of a gene encoding GFP fused to Cre recombinase. It is highly and specifically expressed in erythroid cells. *Sox6* RNA was undetectable in the bone marrow and spleen of 10-week-old *Sox6^fl/fl^ErGFPCre* mice, proving efficient inactivation of *Sox6* in erythroid cells (Fig. 1B). These mice looked externally normal at all ages and complete blood cell counts (CBC) revealed that most of their blood parameters were normal (Fig. 1C; BD and VL, unpublished data). Noticeably, however, their hemoglobin level, hematocrit, and RBC mean cell volume were 5 to 13% lower than normal; they had a 16% increase in red cell distribution width, reflecting RBC anisocytosis, and a 47% increase in reticulocyte count, revealing reticulocytosis; moreover, their serum Epo level (sEpo) was 183% as high as normal, and their spleen was 152% as large as normal. In standard colony-forming assays in vitro, mutant spleen cells generated 32% more erythroid colony-forming units (CFU-Es) than wild-type cells and, although they were slower than controls, they eventually generated 50% more erythroid burst-forming units (BFU-Es) than controls (Fig. 1D). These data thus indicated that *Sox6* ablation in erythroid cells results in compensated anemia in adult mice.

To uncover the cause of this phenotype, we analyzed erythropoietic tissues by FACS. We first used antibodies against CD71 (transferrin receptor) to label late proerythroblasts and erythroblasts, and antibodies against TER119 (glycophorin A-associated protein) to label erythroblasts (Fig. 1E). Mutant bone marrow and spleen tissues showed identical phenotypes, with 25–28% fewer non-/early erythroid cells (CD71^−^/TER119^−^) than control tissues, and a 35–38% increase in the ratio of erythroblasts (CD71^+^/TER119^+^) to late proerythroblasts (CD71^+^/TER119^−^). Using an antibody against the CD44 cell adhesion glycoprotein [44], we found that the size of two populations of developing erythroid cells was significantly altered in mutant tissues: the proerythroblast population (CD44^high^/TER119^−^) and the erythroblast population (CD44^med^/TER119^+^) (Fig. 1F). The population of non-erythroid and erythroid precursor cells (CD44^−/med^/TER119^−^) appeared unchanged, and we therefore used it to calculate that the population of proerythroblasts was depleted by 33%, whereas the population of erythroblasts was more affected, with an enlargement of 60%. We further analyzed the erythroblast population using the thiazole orange (TO) nucleic acid dye to measure chromatin condensation and enucleation, and using the cell forward scatter to measure cell condensation. We thereby found that the increased proportion of erythroblasts in mutant tissues was due to a 3-fold increase in the ratio of uncondensed (FSC^high^/TO^high^) versus condensed erythroblasts (FSC^low^/TO^med^), i.e., early versus late erythroblasts (Fig. 1G). We reached the same conclusion when we analyzed the cells using their FSC and CD44 antibodies instead of TO (data not shown). These data thus demonstrated that *Sox6* is needed in adult mice to facilitate the development of erythroid cells. It is importantly needed at the proerythroblast stage and critically needed at the erythroblast stage.

We then analyzed circulating RBCs. We observed that mature RBCs (TO^−^/CD71^−^) accounted for more than 95% blood cells in control mice, as expected, but for only 70% blood cells in mutant mice (Fig. 1H). In addition, while 67% control reticulocytes (CD71^+^ and/or TO^+^) were retaining RNA longer than CD71 (TO^+^/CD71^low/−^), 81% mutant reticulocytes were maintaining CD71 longer than RNA (TO^−^/CD71^+^). Reticulocytes were thus maturing slowly and abnormally in mutant mice.

Finally, decay profiling of biotin-labeled cells revealed that RBCs had a half-life reduced to 19 days in mutant mice compared to 25 days in control mice (Fig. 1I). We concluded that adult mice maintained under physiological conditions but lacking *Sox6* in erythroid cells exhibited compensated anemia, with underdevelopment of proerythroblasts, impaired maturation of erythroblasts and reticulocytes, and decreased survival of RBCs.

### Impaired erythroid development is a primary cause of anemia upon *Sox6* inactivation

**Figure 2:**
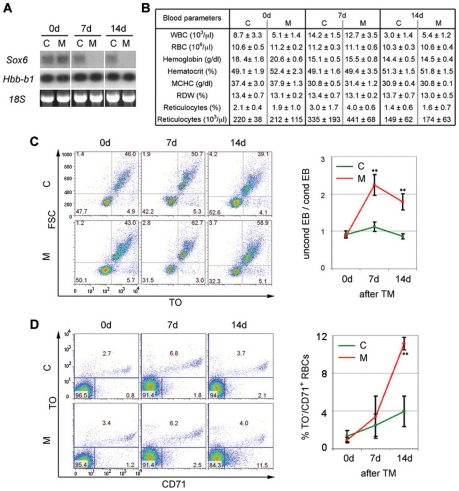
*Sox6* is primarily needed for erythroid cell maturation under adult physiological conditions. (**A**) Northern blot of bone marrow RNA collected 0, 7 and 14 days after initiation of TM treatment of 10-week-old *Sox6^fl/fl^* control (C) and *Sox6^fl/fl^CaggCreER* mutant (M) mice. *Sox6* RNA was detected using a coding-exon-2 probe. Hybridization with an *Hbb-b1* probe and staining with ethidium bromide of 18S rRNA are shown as controls for RNA loading. (**B**) Blood parameters in similar mice as in panel (A). Data are reported as average values with standard deviation obtained for 3 mice per genotype. Other blood parameters measured by the ADVIA instrument and not shown in this table were normal in mutant mice. (**C**) FACS analysis of erythroblasts in the bone marrow of mice treated as in panel (A). Left, representative FSC/TO FACS profiles of TER119^+^/CD71^+^ cells. Right, ratios of uncondensed/condensed erythroblasts. The data are presented as the average with standard deviation of values obtained for 3 mice per genotype. (**D**) FACS analysis of blood from the same mice as in panel (C). Left, representative TO/CD71 FACS profiles. Right, ratios of atypical reticulocytes (TO^−^/CD71^+^). *p<0.05, **p<0.01.

We next tested whether the abnormal development of erythroid cells in *Sox6^fl/fl^ErGFPCre* mice was a direct consequence of *Sox6* inactivation or was due to anemia-induced stress erythropoiesis as a result of reduced survival or malfunction of RBCs. We generated *Sox6^fl/fl^* adult mice harboring a widely expressed *CaggCreER* transgene [36]. This transgene encodes a fusion of Cre recombinase with a mutant estrogen ligand-binding domain, which exclusively binds tamoxifen (TM). The CreER protein is thus able to recombine genes only when mice are treated with TM. *Sox6^fl/fl^CaggCreER* mice showed no visual sign of disease before and for at least one month after TM injection (BD and VL, unpublished data). The *Sox6* RNA level was normal in bone marrow and spleen before treatment, and became undetectable 7 and 14 days after treatment (Fig. 2A). CBC parameters remained normal for at least 2 weeks (Fig. 2B; and, BD and VL, unpublished data). The ratio of uncondensed versus condensed erythroblasts doubled within the first week (Fig. 2C), but abnormal reticulocytes appeared in the circulation only in the second week (Fig. 2D). Developing erythroid cells thus became abnormal before the reduced lifespan or other defects of mutant RBCs became consequential, indicating that impaired erythroid cell maturation is a primary cause of anemia upon *Sox6* inactivation.

### *Sox6* is critically needed for efficient stress erythropoiesis

**Figure 3:**
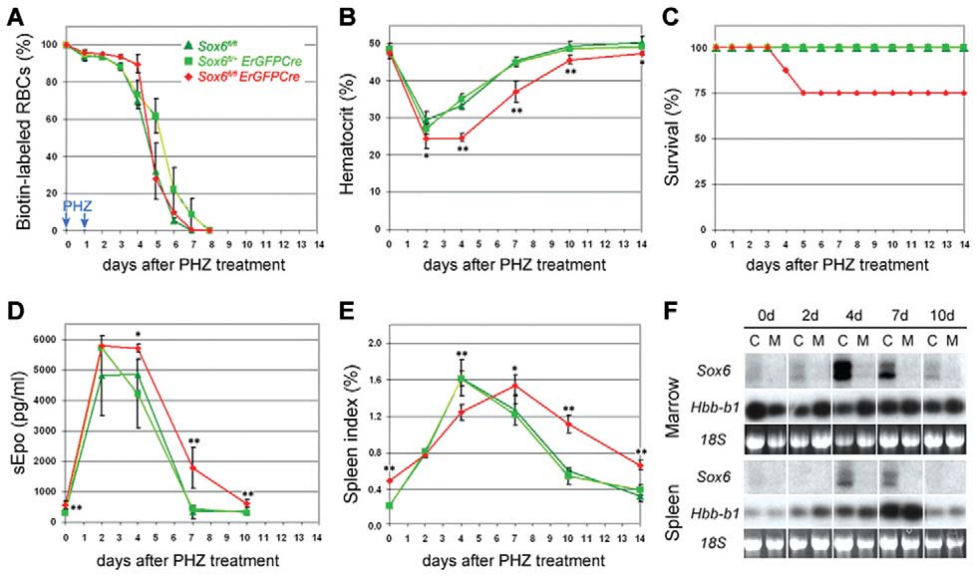
*Sox6* is needed in the stress erythropoietic response to acute anemia. (**A**) Kinetics of RBC decay in *Sox6^fl/fl^* and *Sox6^fl/+^ErGFPCre* control and *Sox6^fl/fl^ErGFPCre* mutant mice over a period of 2 weeks following PHZ treatment. RBCs were labeled with biotin at day –1 and PHZ was injected at the days 0 and 1. The data are presented as the average with standard deviation of values obtained for 3 mice per genotype. (**B**) Kinetics of hematocrit changes in control and mutant mice following PHZ treatment. Each data is the average with standard deviation of values obtained for 3 mice. Different mice were used at each time point. (**C**) Mouse survival curves following PHZ treatment. At day 3, each experimental group contained 24 mice. All mice survived in the control groups, whereas 3 mice died at day 4, and 3 at day 5 in the mutant group. (**D**) Kinetics of sEpo level in the same mice as in panel (B). (**E**) Kinetics of spleen index in the same mice as in panel (B). (**F**) Northern blot analysis of *Sox6* RNA level in the bone marrow and spleen of *Sox6^fl/fl^* control and *Sox6^fl/fl^ErGFPCre* mutant mice at various time points following PHZ treatment. *Sox6* RNA was detected using a coding-exon-2 probe. Hybridization with an *Hbb-b1* probe and staining with ethidium bromide of 18S rRNA are shown as controls for RNA loading. *p<0.05, **p<0.01.

The relatively mild erythroid defects of *Sox6^fl/fl^ErGFPCre* adult mice in basal conditions prompted us to determine whether this phenotype would be exacerbated in stress erythropoiesis. We induced acute anemia by injecting *Sox6^fl/fl^* and *Sox6^fl/+^ErGFPCre* control mice and *Sox6^fl/fl^ErGFPCre* mutant mice with phenylhydrazine (PHZ), a powerful oxidative agent [37]. As expected, all pre-existing RBCs decayed in control mice within a week (Fig. 3A). A similar decay was observed in mutant mice, demonstrating similar sensitivity of mutant and control RBCs to PHZ. The hematocrit of all mice decreased by half within 2 days (Fig. 3B). Control mice started to recover by day 4 and all survived and regained a normal hematocrit by day 10 (Fig. 3B and C). In contrast, mutant mice maintained a low hematocrit for at least two more days and 25% of them died at day 4 or 5. The survivors started to recover by day 7, but their hematocrit was still 10–15% lower than normal at day 10.

A quick, massive surge in sEpo level is a critical, early response to acute anemia. This surge occurred similarly in control and mutant mice and led to a maximal sEpo level from day 2 to day 4 (Fig. 3D). The sEpo level returned to its basal value by day 7 in control mice, but only by day 10 in mutants (Fig. 3D). Hence, the sEpo level was not responsible for the impaired recovery of mutants. Stress erythropoiesis also involves hyperplasia of the spleen, due to erythropoietic burst. Control spleens enlarged about 8 times by day 4 and returned to their original size after day 10 (Fig. 3E). Mutant spleens reached the same final size several days later despite a larger original size and normal surge in sEpo level, and returned to their original size by day 14 only. *Sox6* thus critically participates in the erythropoietic burst generated in response to acute anemia. Supporting this conclusion, the *Sox6* RNA level strongly increased in control bone marrow and spleen by day 4 and through day 7, and remained undetectable in mutant tissues (Fig. 3F). Interestingly, the *Sox6* RNA level increased as the sEpo level surge was fading, but it coincided with the time of death of mutant mice and with the delay in enlarging the spleen and regaining a normal hematocrit. *Sox6* thus participates in stress erythropoiesis mostly beyond the Epo-dominated early phase.

### *Sox6* ensures timely maturation of erythroid cells in stress erythropoiesis

**Figure 4:**
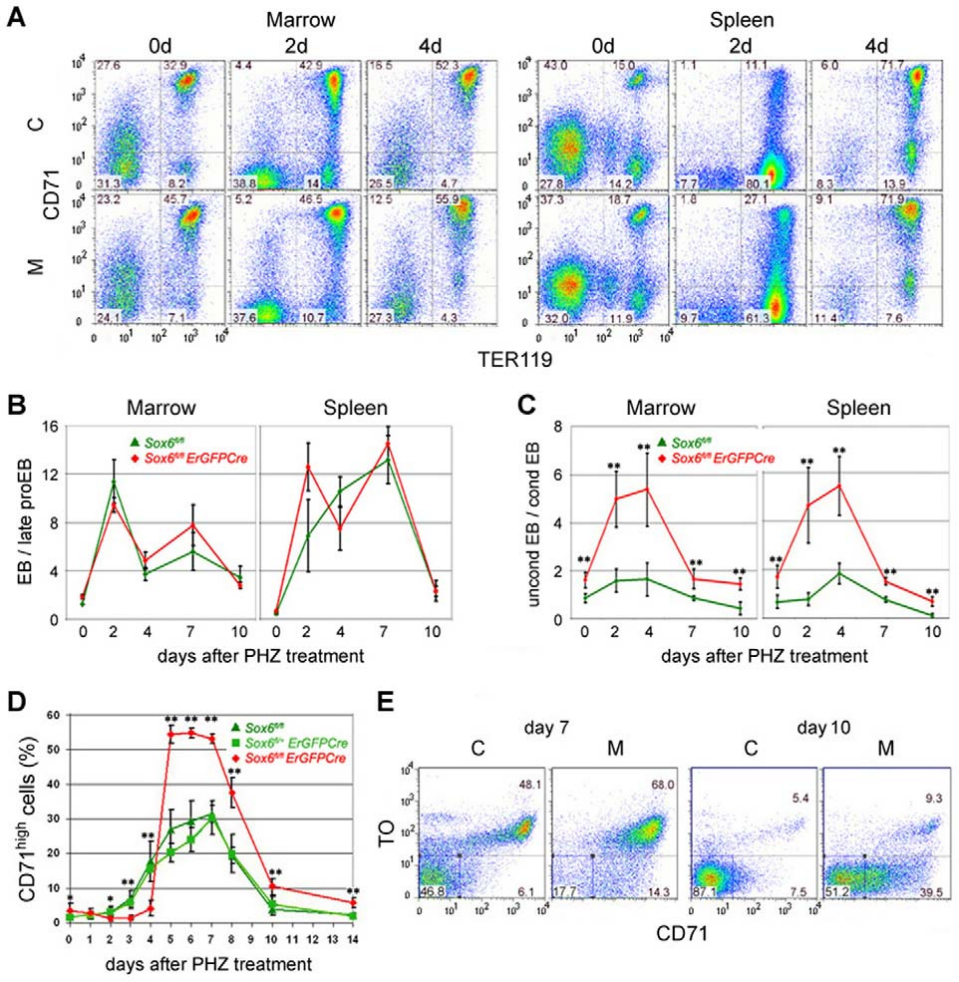
Sox6 ensures quick maturation of erythroblasts and reticulocytes during stress erythropoiesis. (**A**) Representative CD71/TER119 FACS profiles of bone marrow and spleen from *Sox6^fl/fl^* control (C) and *Sox6^fl/fl^ErGFPCre* mutant (M) mice 0, 2, and 4 days after PHZ treatment. Note that a massive increase in CD71^−^/TER119^+^ cells occurred in control and mutant spleens at day 2, reflecting clearance of damaged RBCs. (**B**) Kinetics of the proportions of erythroblasts versus proerythroblasts in bone marrow and spleen of control and mutant mice from day 0 to day 10 following PHZ treatment. Values were derived from CD71/TER119 FACS profiles and are presented as the average with standard deviation for 3 mice per genotype at each time point. (**C**) Kinetics of the proportions of uncondensed versus condensed erythroblasts. Values were derived from FSC/TO FACS profiles performed with the same cell populations as in panel (B). (**D**) Kinetics of the percentage of reticulocytes (CD71^high^) present in the blood of control and mutant mice following PHZ treatment. Values are presented as the average with standard deviation for at least 3 mice per genotype at each time point. (**E**) Representative TO/CD71 FACS profiles of blood cells from control and mutant mice 7 and 10 days after PHZ treatment. *p<0.05, **p<0.01.

To identify the contribution of *Sox6* in stress erythropoiesis, we analyzed erythropoietic tissues and blood samples of PHZ-treated *Sox6^fl/fl^* and *Sox6^fl/fl^ErGFPCre* mice. From day 2 to day 7 post treatment, control and mutant mice increased the proportion of erythroblasts (CD71^+^/TER119^+^) versus late proerythroblasts (CD71^+^/TER119^−^) 10–20 fold in bone marrow and spleen (Fig. 4A and B). Interestingly, the proportion of uncondensed to condensed erythroblasts increased up to 2 fold in control mice and up to 5 fold in mutant mice under stress erythropoiesis (Fig. 4C). *Sox6* thus ensures speedy maturation of erythroblasts in both basal and stress erythropoiesis.

Reticulocytes could not be detected in the week that followed PHZ injection, likely due to interference of PHZ with RNA dyes (BD and VL, unpublished data). We therefore assayed reticulocytes using CD71 antibody only. The quick recovery of control mice from acute anemia could be explained by an increase in reticulocyte percentage that became significant by as early as day 3 and that remained at 20–30% between day 5 and day 8 before returning to normal by day 10 (Fig. 4D). In contrast, the inability of mutant mice to quickly recover could be explained by failure to increase the reticulocyte percentage before day 5. Surprisingly, this parameter became almost twice as high as in control mice from day 5 and remained elevated until mice fully recovered. By day 7, when all RBCs were less than 1-week old (Fig. 3A), almost 50% of them were already mature in control mice, but less than 20% were mature in mutants (Fig. 4E). By day 10, about 90% RBCs were mature in control mice, but only about 50% were mature in mutants. *Sox6* thus critically intervenes in stress erythropoiesis to ensure prompt maturation of erythroblasts and reticulocytes.

### Sox6 upregulates expression of *Bcl2l1* and other erythroid terminal markers

**Figure 5:**
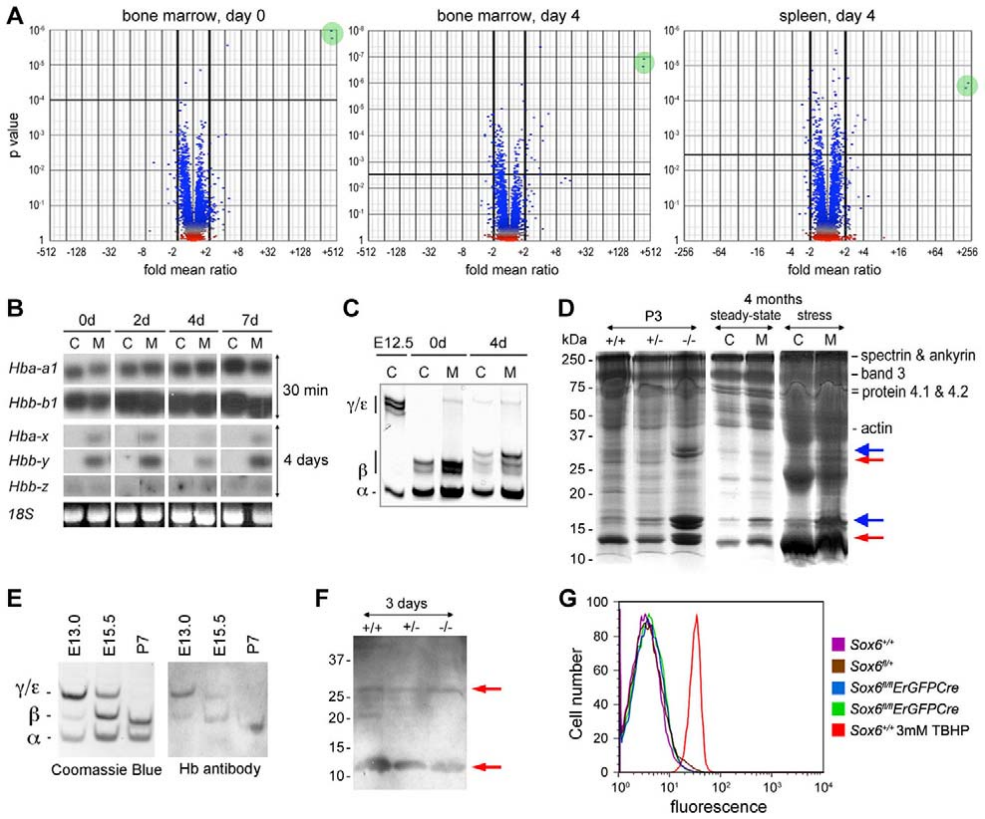
Embryonic globin and other gene expression changes in *Sox6*-deficient erythropoietic tissues and primary erythroid cells. (**A**) Volcano plots showing gene expression changes in mutant tissues. RNA was from bone marrow of *Sox6^fl/fl^* and *Sox6^fl/fl^ErGFPCre* mice before PHZ treatment (day 0) [left]; bone marrow [middle] and spleen [right] of similar mice 4 days after treatment. Each dot represents a gene probe. Green circles highlight *Hbb-y* identified by two probes. (**B**) Northern blot of globin RNA levels in spleen of *Sox6^fl/fl^* (C) and *Sox6^fl/fl^ErGFPCre* (M) mice 0 to 7 days after PHZ treatment. Probes were for adult alpha-globin (*Hba-a1*) and beta-globin (*Hbb-b1*), and embryonic X-globin (*Hba-x*), Y-globin (*Hbb-y*), and Z-globin (*Hbb-z*). All had similar specific activity and length. X-ray films were exposed for 30 min for adult globin RNAs, and 4 days for embryonic globin RNAs. The latter were thus weakly expressed compared to the former. (**C**) Electrophoretic analysis of globins in RBCs from E12.5 wild-type embryo and control and mutant adult mice 0 and 4 days after PHZ treatment. Globin chains are indicated. Several beta-globin isoforms are seen due to mixed genetic background. (**D**) RBC ghosts from 3-day-old (P3) *Sox6^+/+^*, *Sox6^+/−^*, and *Sox6^−/−^* littermates, and 4-month-old *Sox6^fl/fl^* (C) and *Sox6^fl/fl^ErGFPCre* (M) mice 0 day (steady-state) and 4 days (stress erythropoiesis) after PHZ treatment. Protein marker molecular weights and typical RBC proteins are indicated. Blue arrows, ectopic histones in mutants. Red arrows, proteins reacting with hemoglobin antibody against beta-, gamma-, delta-, and epsilon-globins. (**E**) Coomassie blue staining of globins in RBC lysates from control mice at E13.0, E15.5, and postnatal day 7 (P7) and western blot with hemoglobin antibody. (**F**) Western blot of ghosts from P3 mice with hemoglobin antibody. Red arrows, all samples have similar residual globin chains. (**G**) Flow cytometry shows that minimal ROS production in blood of control and mutant adult mice, except in control blood treated with *tert*-butyl hydroperoxide (TBHP).

To better understand how *Sox6* controls erythroid cell development, we screened gene expression microarrays with bone marrow and spleen RNA from *Sox6^fl/fl^* and *Sox6^fl/fl^ErGFPCre* littermates at 3 months of age under physiological conditions and 4 days after induction of acute anemia by PHZ. Volcano plots of p values against gene expression fold changes revealed that the embryonic epsilon globin gene (*Hbb-y*) was by far the most affected gene, with an upregulation fold of more than 200 in all mutant samples (Fig. 5A).

*Hbb-y* was previously shown to be a direct target of Sox6 in erythroid cells [31], [32], but it was not shown whether its ectopic expression impaired the development of erythroid cells. We tested this possibility because imbalanced production of globin chains in thalassemia results in impaired maturation and survival of erythroid cells in a manner resembling the *Sox6*-null phenotype [45], [46]. Northern blot analysis revealed that the embryonic globin genes were expressed at a much lower level than adult globin genes in *Sox6*-null erythroid cells (Fig. 5B). Accordingly, embryonic globin chains were hardly if at all detected in RBC lysates (Fig. 5C). A detrimental consequence of the imbalanced production of globin chains in thalassemia is precipitation of globin chains in erythroid membranes. Precipitated chains can be detected in ghosts, i.e., red blood cell membrane extracts. *Sox6*-null ghosts contained ectopic proteins with an Mr close to 15 and 30k (Fig. 5D). These proteins, however, did not react with an antibody against beta, gamma, delta and epsilon globins (Fig. 5E and F), and mass spectrometry analysis revealed that the 15k and 30k species largely corresponded to H2a/2b and H1 histones, respectively, and did not contain any globin chain (PD and VL, unpublished data). These histones thus reflected the fact that *Sox6* mutant mice release a significant proportion of nucleated erythroid cells in the circulation [30]. Another consequence of the abnormal production of globin chains in thalassemia is the generation of reactive oxygen species (ROS) [47]. ROS are very detrimental to cells, causing protein damage and premature death. Their production was not increased in the blood of *Sox6*-null mice (Fig. 5G). Together, these data thus indicated that the abnormal erythroid phenotype of *Sox6* mutant mice is unlikely due to ectopic expression of embryonic globin genes.

**Figure 6:**
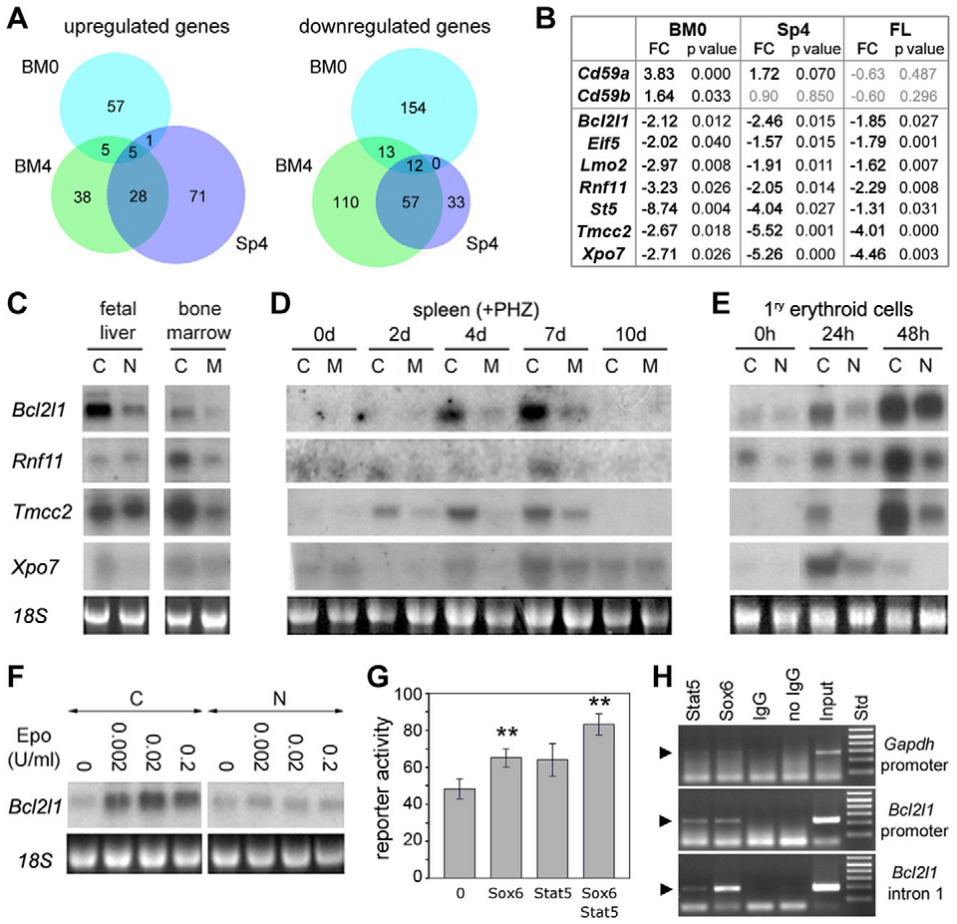
Characterization of *Bcl2l1* and other gene expression changes in *Sox6*-deficient tissues and cells. (**A**) Venn diagram showing the number of genes upregulated or downregulated at least 1.5 fold (*p≤0.1*) in *Sox6* mutant samples. BM0, bone marrow before PHZ treatment; BM4, bone marrow 4 days after PHZ treatment; Sp4, spleen 4 days after PHZ treatment. (**B**) Real-time RT-PCR validation of a subset of genes significantly upregulated or downregulated in *Sox6* mutant samples in microarrays. At least three mice were used for each type of samples. FC, fold change. (**C**) Northern blot of total RNA from the liver of *Sox6^+/+^* (C) and *Sox6^−/−^* (N) littermates at E14.5, and from the bone marrow of *Sox6^fl/fl^* (C) and *Sox6^fl/fl^ErGFPCre* (M) adult mice under physiological conditions. Hybridization was with the indicated probes. (**D**) Northern blot of total RNA from the spleen of *Sox6^fl/fl^* (C) and *Sox6^fl/fl^ErGFPCre* (M) mice 0 to 10 days after PHZ treatment. (**E**) Northern blot of total RNA from TER119^−^ liver cells isolated from E14.5 control (C) and *Sox6^−/−^* (N) fetuses and cultured in erythroid differentiation medium for 0, 24, and 48 h. Most cells are at the early or late erythroblast stage by 24 and 48 h, respectively [30]. (**F**) Northern blot of total RNA from TER119^−^ liver cells isolated from E14.5 control (C) and *Sox6^−/−^* (N) fetuses and cultured in erythroid differentiation medium for 24 h with the indicated Epo concentrations. (**G**) Transient transfection of Cos-1 mesenchymal cells with a *Bcl2l1* construct and expression plasmids for Sox6 and constitutively active Stat5. Assays were performed in triplicates and are presented as the average with standard deviation for one representative experiment. **p<0.01. (**H**) Chromatin immunoprecipitation assay in mouse fetal liver. Chromatin was precipitated with the indicated antibodies. PCR was performed with immunoprecipitated and input chromatin fragments using primers to amplify the *Gapdh* promoter (negative control), and the *Bcl2l1* promoter and intron 1.

Besides *Hbb-y* upregulation, most other gene expression changes were less than 4-fold in *Sox6* mutant samples (Fig. 5A). Few genes had their expression changed under both steady-state and erythropoietic stress conditions, but many genes had their expression affected in both bone marrow and spleen under stress erythropoiesis (Fig. 6A and Table S1). We selected a subset of upregulated and downregulated genes according to their known or predicted functions and fold expression change, and validated their expression changes by quantitative RT-PCR using RNA from fetal liver and from samples similar to those used for the microarrays. We confirmed that the genes for CD59a and CD59b, which restrict the lysis of human erythrocytes and leukocytes by the homologous complement, tended to be upregulated in the bone marrow and spleen of *Sox6* mutant mice (Fig. 6B). All selected downregulated genes were confirmed in this assay and in northern blot (Fig. 6B and C). They included *Bcl2l1*, which encodes the anti-apoptotic factor Bcl-xL; *Eif5*, which encodes the eukaryotic translation initiation factor 5; *Lmo2,* which codes for the LIM-domain only factor 2 and is critically needed for hematopoietic development; *Rnf11,* which codes for the E3 ubiquitin ligase ring finger factor 11; *St5,* which codes for the suppressor of tumorigenicity 5 factor; *Tmcc2,* the gene for the transmembrane and coiled-coil domain family 2; and *Xpo7*, the gene for exportin 7. Most of these genes were upregulated at day 4 and 7 during stress erythropoiesis in wild-type mice, coincidentally with erythroid terminal maturation and *Sox6* expression (Fig. 3F and 6D). They were also upregulated when wild-type erythroid cells were reaching the early and late erythroblast stages at 24 and 48 h in primary cultures, respectively (Fig. 6E). These data thus indicated that Sox6 is needed to upregulate expression of late erythroid markers.

*Bcl2l1* was previously shown to mediate erythroid cell survival and to be a direct target of the transcription factor Stat5 downstream of Epo signaling [19]–[21]. Interestingly, we found that upregulation of *Bcl2l1* expression in response to Epo signaling required *Sox6* (Fig. 6F). Furthermore, Sox6 was able to upregulate a *Bcl2l1* reporter containing the most highly conserved non-coding regions of the gene, including an upstream enhancer, the proximal promoter and first intron (Fig. 6G). These regions were previously shown to control *Bcl2l1* expression in erythroid cells in response to Epo signaling [21], [43]. Sox6 was as potent as constitutively active Stat5 in upregulating the activity of the *Bcl2l1* reporter, and the two factors had additive effects. Additionally, a chromatin immunoprecipitation assay in wild-type fetal liver cells provided evidence that Sox6, like Stat5, directly binds to the promoter and first intron of the endogenous *Bcl2l1* gene in erythroid cells in vivo (Fig. 6H). These data thus strongly suggest that *Bcl2l1* is a direct target of Sox6 downstream of Epo signaling and during erythroid terminal maturation.

## Discussion

This study has uncovered that Sox6 is expressed in erythroid cells throughout life and acts as a significant enhancer of both basal and stress erythropoiesis in the adult mouse. Sox6 has important roles in the development of proerythroblasts and also has critical roles in promoting maturation of erythroblasts and reticulocytes and in extending the life span of RBCs. Its ability to repress the embryonic globin genes appears inconsequential. In contrast, its ability to directly enhance *Bcl2l1* expression likely contributes to its ability to work with and beyond Epo signaling in promoting erythroid cell survival.

We demonstrated that *Sox6* significantly contributes to ensuring efficient basal erythropoiesis by showing that *Sox6^fl/fl^ErGFPCre* adult mice had compensated anemia. Clear signs of compensation included an increase of up to 2 fold of the reticulocyte count, sEpo level, spleen size, and a 32–50% increase in the number of erythroid colonies developing in vitro from spleen cells. It is very likely that erythroid defects are the cause of this anemia since wild-type adult mice strongly express *Sox6* in erythropoietic tissues and since *Sox6^fl/fl^ErGFPCre* mice inactivate *Sox6* only in erythroid cells. Further supporting this mechanism, we showed that *Sox6^fl/fl^ErGFPCre* mice had erythroid cell defects and decreased RBC life span, and that *Sox6^fl/fl^CaggCreER* adult mice, which inactivated *Sox6* only upon tamoxifen injection, developed erythroblast defects before anemia. The shorter half-life of RBCs must have contributed to cause anemia, but its value at 19 instead of 25 days was probably insufficient to justify the doubling of the sEpo level and spleen size. It is likely that abnormal maturation of RBCs reduced the functionality of these cells and thus contributed to causing hypoxia. The enlargement of the spleen could have several causes. We demonstrated that it was partly due to increased in erythropoietic activity, since more erythroid colonies could be generated in vitro from mutant spleens than from control spleens. We showed that it was also due to a larger population of erythroblasts. As previously reported for fetal liver erythroid cells [30], erythroblasts from adult bone marrow and spleen were impaired in maturing and thus delayed in entering the circulation. Interestingly, we found that the erythropoietic tissues of adult mutants had a reduced proportion of proerythroblasts, revealing that *Sox6* is needed to promote expansion of proerythroblasts. We previously reported that *Sox6* ablation in fetal liver primary erythroid cells resulted in decreased survival and proliferation of proerythroblasts [30]. Similar defects in proerythroblasts are thus likely to occur in *Sox6* mutant adult mice. They would explain that erythropoietic tissues contained fewer proerythroblasts and that BFU-Es formed more slowly from mutant than from wild-type adult erythropoietic tissues in vitro. Our new data thus demonstrate that Sox6 enhances definitive erythropoiesis in a similar way in the fetus, young pup, and adult mouse. While this is not entirely surprising, we could not have extrapolated the conclusion previously drawn in young animals to adults without experimentation since erythropoiesis occurs at a much lower rate in the adult under steady-state conditions than in the rapidly growing fetus and pup, and since major differences exist in erythroid gene expression between fetuses and adult mice [48], [49].

We demonstrated that *Sox6* is more critically needed in adult mice subjected to stress erythropoiesis than in mice under steady-state conditions of erythropoiesis. Similar conclusions were previously reached for other factors, such as Bcl-xL and Foxo3a, and is explained by the fact that stress erythropoiesis is a much more productive process than basal erythropoiesis and thus critically relies on factors capable of boosting it. *Sox6^fl/fl^ErGFPCre* mice showed a mortality of 25% when subjected to acute anemia, whereas all control mice survived. RBCs decayed at the same rate in control and mutant mice and the sEpo level increased similarly in both types of mice, ruling out increased sensitivity of mutants to PHZ or inability to generate a sufficient surge in Epo level. The hematocrit of control and mutant mice decreased by half within 2 days of PHZ injection, but remained at this low value much longer in mutant than control mice. Such a low value is not life threatening in a normal mouse. While the hematocrit of the mutant mice that died could have further dropped, additional defects could also have contributed to lethality. Reticulocytes were maturation impaired, maintaining a high level of CD71 longer than control cells. We tried to measure hemoglobin levels in the first week after PHZ treatment, but the high degree of hemolysis at that time did not permit accurate measurement. We previously showed that the RBC hemoglobin content of *Sox6^−/−^* fetuses was about 66% the normal value and that *Sox6^−/−^* primary erythroid cells were delayed in hemoglobinizing [30]. It is thus possible that the first RBCs released in mutant mice under stress were poorly hemoglobinized. Moreover, their immature shape and possibly other defects may have further reduced their ability to properly deliver oxygen to tissues.

We showed that *Sox6* has unique roles in stress erythropoiesis. *Sox6* is dispensable to generate the surge in sEpo level and the Epo-mediated early response to acute anemia, which includes spleen enlargement and massive production of proerythroblasts and early erythroblasts. Further supporting the notion that *Sox6* is not involved in this early response was our observation that *Sox6* expression increases in bone marrow and spleen only from day 4 to 7 after PHZ injection. We previously showed that *Sox6* enhances the ability of Epo signaling to promote proerythroblast survival and proliferation in vitro [30] and we showed here that Sox6 is needed probably in the same way to expand the population of proerythroblasts in adult mice under physiological conditions. This function of Sox6, however, is probably largely compensated for in mutant mice subjected to acute anemia by the very high increase in sEpo level. In contrast, the major role of *Sox6* to promote erythroblast maturation in the second phase of the stress erythropoietic response, including cytoplasmic and nuclear condensation, is not fully compensated. Such defects were seen under basal conditions, but they were worsened under stress conditions. *Sox6* thus most critically control erythroid cells at the terminal maturation stages at all mouse ages in both basal and stress conditions for erythropoiesis.

We provided new insights into the mechanisms whereby *Sox6* controls erythroid development at the molecular level by screening gene expression microarrays and validating data with various RNA and protein assays. Previous studies in fetuses and hematopoietic chimeras revealed that the embryonic globin genes are ectopically expressed in *Sox6*-deficient definitive erythroid cells [30]–[33]. We have extended these findings to adult mice in both basal and stress erythropoiesis conditions. Importantly, we have also demonstrated that the globin RNA and protein levels are negligible compared to those of adult globins and that these proteins do not precipitate in the erythroid membranes and do not increase the production of damaging reactive oxygen species, in contrast that what is seen when abnormal ratios of globin proteins are produced in thalassemia patients. Repression of embryonic globin genes is thus unlikely to be the most consequential function of *Sox6* in erythroid cells. We found that many other genes were upregulated or downregulated in the absence of *Sox6*. The expression changes of these genes were only a few folds, a finding consistent with the fact that Sox6 does not have a transactivation or transrepression domain, and has been shown to act in other cell types as a positive or negative modulator of gene expression [23]. One of the downregulated genes was *Bcl2l1*, which encodes the anti-apoptotic factor Bcl-xL. Previous studies have shown that *Bcl2l1* is a direct target of Stat5 downstream of Epo signaling in early erythroid cells [21], and that it is upregulated in late erythroid cells in a Stat5-independent manner to promote erythroblast and reticulocyte survival [22], [50]–[52]. In agreement with these studies, we showed that *Bcl2l1* expression closely parallels *Sox6* expression in late erythroid cells and is dependent upon both Epo signaling and Sox6. Further, we demonstrated that both Sox6 and Stat5 are able to upregulate a *Bcl2l1* reporter in cultured cells, and their effects are additive rather than synergistic. This result is consistent with the possibility that Stat5 and Sox6 both contribute to mediate Epo signaling in vivo but act independently of each other rather than cooperatively. While Stat5 is more critical in early erythroid cells, Sox6 is more critical at later stages of erythroid cell development. Further studies in vivo and in vitro will be needed to fully decipher how Stat5 and Sox6 contribute to control *Bcl2l1* expression at the various stages of erythroid cell development and in response to Epo signaling. Another interesting gene significantly downregulated in *Sox6* mutants is *Rnf11*. Although its role in vivo remains unknown, it is tempting to speculate that the E3 ubiquitin ligase activity of its ring finger protein 11 product [53] may contribute to the important roles that ubiquitination fulfills in erythroid cells. It has been shown, for instance, that ubiquitination causes rapid inactivation of EpoR upon ligand binding [54] and participates in reticulocyte remodeling, including CD71 sorting into exosomes [55]. Poor ubiquitination could thus account for several defects in *Sox6* mutants, including retention of CD71 on reticulocytes. Together, these results thus strongly suggest that Sox6 facilitates erythroid cell development by modulating the expression of a large panel of marker genes.

Definitive RBCs are more efficient than primitive RBCs because they are small, lack a nucleus, contain adult rather than embryonic globins, and are long-lived. We have shown here that Sox6 acts throughout adult life to facilitate erythropoiesis. It is important in both basal and stress erythropoiesis. It ensures that proerythroblasts properly expand and that RBCs are long-lived, but its most critical roles are to ensure that the output of definitive erythropoiesis is not delayed or decreased by the additional steps of cell maturation required for erythroblast condensation and enucleation. In addition to Epo treatments widely used to generate an adequate burst of early erythroid cells in patients with various anemia-causing conditions, one should thus also consider targeting Sox6 to ensure timely maturation and release, as well as long life of newly generated red blood cells.

## Supporting Information

Table S1 List of gene expression changes in Sox6 mutant samples versus control samples, as determined by microarray screening.
(0.98 MB DOC)
