# Supplementary material for: Sox6 Is Necessary for Efficient Erythropoiesis in Adult Mice under Physiological and Anemia-Induced Stress Conditions
Source: PLoS One. 2010 Aug 9;5(8):e12088. doi: 10.1371/journal.pone.0012088 (PMC2918505; doi:10.1371/journal.pone.0012088)
Supplement: Table S1 — List of gene expression changes in Sox6 mutant samples versus control samples, as determined by microarray screening. (0.98 MB DOC) [file pone.0012088.s001.doc]

| **Genes upregulated ≥1.5 fold. P value ≤0.1** | | | | | | | | |
| --- | --- | --- | --- | --- | --- | --- | --- | --- |
|  | | | | | | | | |
| **All samples** | | | **BM day 0** | | **BM day 4** | | **Sp day 4** | |
|  | *Gene symbol* | | *fold change* | *p value* | *fold change* | *p value* | *fold change* | *p value* |
| 1 | *Hbb-y* | | -419.809 | 1.71E-06 | -386.506 | 1.23E-07 | -228.252 | 3.13E-05 |
| 2 | *Xk* | | -4.321 | 2.76E-06 | -4.004 | 4.23E-08 | -1.998 | 7.17E-04 |
| 3 | *Tuft1* | | -2.524 | 2.44E-02 | -4.171 | 4.41E-05 | -3.167 | 8.22E-03 |
| 4 | *Slc30a10* | | -2.001 | 2.75E-02 | -2.046 | 5.24E-04 | -1.630 | 7.11E-02 |
| 5 | *Ppp1r9b* | | -1.686 | 4.39E-04 | -1.893 | 4.84E-06 | -1.536 | 2.24E-03 |
|  | | | | | | | | |
| **BM days 0 and 4** | | | **BM day 0** | | **BM day 4** | |  | |
|  | *Gene symbol* | | *fold change* | *p value* | *fold change* | *p value* |  |  |
| 1 | *Cd59a* | | -3.703 | 3.14E-02 | -3.003 | 1.62E-02 |  |  |
| 2 | *OTTMUSG00000016790* | | -2.957 | 1.67E-02 | -2.096 | 4.65E-02 |  |  |
| 3 | *Cited4* | | -2.345 | 1.33E-02 | -2.632 | 6.88E-04 |  |  |
| 4 | *Golga2* | | -1.926 | 4.87E-02 | -1.949 | 3.64E-02 |  |  |
| 5 | *Tyki* | | -1.591 | 2.66E-02 | -1.658 | 1.52E-02 |  |  |
|  | | | | | | | | |
| **BM day 0 and Sp day 4** | | | **BM day 0** | |  |  | **Sp day 4** | |
|  | *Gene symbol* | | *fold change* | *p value* |  |  | *fold change* | *p value* |
| 1 | *Cd59b* | | -4.100 | 9.77E-04 |  |  | -1.644 | 6.40E-02 |
|  | | | | | | | | |
| **BM and Sp day 4** | | |  | | **BM day 4** | | **Sp day 4** | |
|  | *Gene symbol* | |  |  | *fold change* | *p value* | *fold change* | *p value* |
| 1 | *Pla2g4c* | |  |  | -5.610 | 1.45E-05 | -3.209 | 2.61E-04 |
| 2 | *Fam132b* | |  |  | -4.343 | 1.60E-04 | -4.208 | 3.47E-04 |
| 3 | *Arsg* | |  |  | -3.005 | 2.03E-05 | -2.891 | 5.16E-04 |
| 4 | *Arhgdig* | |  |  | -2.731 | 5.08E-02 | -2.408 | 4.38E-03 |
| 5 | *Pycr2* | |  |  | -2.422 | 1.48E-04 | -2.023 | 1.28E-03 |
| 6 | *Rragd* | |  |  | -2.210 | 3.14E-04 | -1.728 | 1.40E-02 |
| 7 | *Gpr172b* | |  |  | -2.176 | 2.88E-02 | -2.540 | 9.70E-03 |
| 8 | *Cish* | |  |  | -2.152 | 2.09E-03 | -2.261 | 2.01E-02 |
| 9 | *Slc6a13* | |  |  | -2.098 | 2.57E-02 | -2.218 | 2.30E-02 |
| 10 | *Acadsb* | |  |  | -2.044 | 1.02E-05 | -2.145 | 2.25E-05 |
| 11 | *Mllt3* | |  |  | -1.920 | 3.90E-03 | -1.540 | 7.96E-03 |
| 12 | *Gmpr* | |  |  | -1.891 | 4.92E-02 | -2.518 | 1.76E-03 |
| 13 | *Tsc22d2* | |  |  | -1.769 | 1.44E-04 | -1.618 | 4.28E-03 |
| 14 | *Prr13* | |  |  | -1.751 | 6.00E-05 | -2.009 | 1.76E-02 |
| 15 | *Tmem97* | |  |  | -1.687 | 5.31E-03 | -1.645 | 8.25E-04 |
| 16 | *Tnfaip8* | |  |  | -1.645 | 6.41E-03 | -1.836 | 6.19E-02 |
| 17 | *Frrs1* | |  |  | -1.636 | 1.41E-04 | -1.768 | 8.64E-04 |
| 18 | *2310008H09Rik* | |  |  | -1.628 | 5.79E-02 | -1.572 | 1.32E-02 |
| 19 | *Osm* | |  |  | -1.593 | 1.74E-02 | -2.659 | 8.12E-04 |
| 20 | *Rrp12* | |  |  | -1.588 | 9.94E-02 | -1.694 | 1.62E-02 |
| 21 | *Artn* | |  |  | -1.585 | 1.09E-02 | -1.697 | 2.80E-02 |
| 22 | *Trappc4* | |  |  | -1.576 | 3.98E-03 | -1.582 | 2.14E-03 |
| 23 | *1110001J03Rik* | |  |  | -1.557 | 3.13E-02 | -1.530 | 1.03E-03 |
| 24 | *Smn1* | |  |  | -1.532 | 1.23E-02 | -1.621 | 4.68E-03 |
| 25 | *Sssca1* | |  |  | -1.522 | 7.08E-02 | -1.781 | 8.38E-04 |
| 26 | *Noc4l* | |  |  | -1.522 | 6.01E-02 | -1.694 | 1.76E-03 |
| 27 | *Tmem147* | |  |  | -1.519 | 2.13E-02 | -1.597 | 3.46E-04 |
| 28 | *Chchd4* | |  |  | -1.509 | 3.34E-02 | -1.807 | 1.24E-03 |
|  | | | | | | | | |
| **BM day 0** | | | **BM day 0** | |  |  |  |  |
|  | *Gene symbol* | | *fold change* | *p value* |  |  |  |  |
| 1 | *Hist1h2ac* | | -4.637 | 6.38E-02 |  |  |  |  |
| 2 | *Il10* | | -3.393 | 8.36E-02 |  |  |  |  |
| 3 | *Iswim4* | | -3.012 | 1.13E-03 |  |  |  |  |
| 4 | *Axud1* | | -2.573 | 2.46E-02 |  |  |  |  |
| 5 | *Frat2* | | -2.482 | 9.10E-02 |  |  |  |  |
| 6 | *Gpx2* | | -2.429 | 5.83E-02 |  |  |  |  |
| 7 | *Depp* | | -2.230 | 6.03E-02 |  |  |  |  |
| 8 | *Mybpc2* | | -2.207 | 9.73E-02 |  |  |  |  |
| 9 | *Tubb2b* | | -2.162 | 8.81E-03 |  |  |  |  |
| 10 | *Zcchc18* | | -2.106 | 2.93E-02 |  |  |  |  |
| 11 | *Pkhd1l1* | | -2.070 | 8.02E-02 |  |  |  |  |
| 12 | *Trim2* | | -2.046 | 1.69E-03 |  |  |  |  |
| 13 | *Bbc3* | | -1.943 | 7.51E-02 |  |  |  |  |
| 14 | *Cecr5* | | -1.912 | 3.35E-03 |  |  |  |  |
| 15 | *Gnb4* | | -1.905 | 1.12E-02 |  |  |  |  |
| 16 | *Igf2bp3* | | -1.894 | 7.15E-02 |  |  |  |  |
| 17 | *Zfp68* | | -1.883 | 1.01E-02 |  |  |  |  |
| 18 | *Nipsnap1* | | -1.876 | 4.21E-02 |  |  |  |  |
| 19 | *Whrn* | | -1.864 | 8.62E-02 |  |  |  |  |
| 20 | *Hspa2* | | -1.803 | 1.68E-02 |  |  |  |  |
| 21 | *Med25* | | -1.801 | 6.03E-02 |  |  |  |  |
| 22 | *Usp45* | | -1.790 | 7.85E-02 |  |  |  |  |
| 23 | *Prrc1* | | -1.747 | 9.59E-02 |  |  |  |  |
| 24 | *Unc45a* | | -1.737 | 5.91E-02 |  |  |  |  |
| 25 | *CCDC84* | | -1.701 | 8.92E-02 |  |  |  |  |
| 26 | *Slc25a28* | | -1.697 | 8.67E-02 |  |  |  |  |
| 27 | *AI662250* | | -1.689 | 7.26E-02 |  |  |  |  |
| 28 | *Gzf1* | | -1.682 | 8.03E-02 |  |  |  |  |
| 29 | *Pou2f1* | | -1.675 | 3.84E-02 |  |  |  |  |
| 30 | *Mrpl2* | | -1.671 | 2.67E-02 |  |  |  |  |
| 31 | *Ercc5* | | -1.664 | 4.46E-02 |  |  |  |  |
| 32 | *Ero1lb* | | -1.654 | 9.03E-02 |  |  |  |  |
| 33 | *Nfatc3* | | -1.652 | 2.77E-02 |  |  |  |  |
| 34 | *Scd1* | | -1.647 | 9.96E-02 |  |  |  |  |
| 35 | *Sept6* | | -1.646 | 1.54E-02 |  |  |  |  |
| 36 | *Zfp36l1* | | -1.637 | 8.07E-02 |  |  |  |  |
| 37 | *Trp53inp1* | | -1.625 | 1.08E-02 |  |  |  |  |
| 38 | *Utx* | | -1.623 | 6.07E-02 |  |  |  |  |
| 39 | *Slc25a16* | | -1.619 | 7.18E-02 |  |  |  |  |
| 40 | *Rbx1* | | -1.569 | 7.71E-02 |  |  |  |  |
| 41 | *Itpr3* | | -1.568 | 5.21E-02 |  |  |  |  |
| 42 | *Armcx2* | | -1.559 | 7.85E-02 |  |  |  |  |
| 43 | *Evi5* | | -1.555 | 9.83E-02 |  |  |  |  |
| 44 | *Bcl2l11* | | -1.548 | 7.94E-02 |  |  |  |  |
| 45 | *C230071H18Rik* | | -1.545 | 3.56E-03 |  |  |  |  |
| 46 | *Csnk1g2* | | -1.537 | 2.60E-02 |  |  |  |  |
| 47 | *Nelf* | | -1.532 | 1.50E-02 |  |  |  |  |
| 48 | *Btbd1* | | -1.528 | 4.26E-02 |  |  |  |  |
| 49 | *Usp22* | | -1.526 | 6.97E-02 |  |  |  |  |
| 50 | *Csrp2* | | -1.526 | 1.52E-03 |  |  |  |  |
| 51 | *Dusp2* | | -1.525 | 8.31E-02 |  |  |  |  |
| 52 | *Metrn* | | -1.514 | 4.87E-02 |  |  |  |  |
| 53 | *Zmiz2* | | -1.514 | 5.58E-02 |  |  |  |  |
| 54 | *Bzrap1* | | -1.513 | 4.09E-02 |  |  |  |  |
| 55 | *Nab1* | | -1.511 | 4.88E-02 |  |  |  |  |
| 56 | *Atp1b1* | | -1.504 | 4.14E-02 |  |  |  |  |
| 57 | *BC031781* | | -1.502 | 7.86E-02 |  |  |  |  |
|  | | | | | | | | |
| **BM day 4** | | |  | | **BM day 4** | |  | |
|  | *Gene symbol* | |  |  | *fold change* | *p value* |  |  |
| 1 | *Stfa2* | |  |  | -14.703 | 5.25E-03 |  |  |
| 2 | *Stfa3* | |  |  | -11.717 | 4.19E-03 |  |  |
| 3 | *Hbb-bh1* | |  |  | -3.948 | 2.93E-03 |  |  |
| 4 | *Mgp* | |  |  | -3.796 | 1.72E-03 |  |  |
| 5 | *EG433016* | |  |  | -3.077 | 8.19E-04 |  |  |
| 6 | *Asprv1* | |  |  | -2.691 | 1.31E-02 |  |  |
| 7 | *Mmp13* | |  |  | -2.608 | 8.79E-05 |  |  |
| 8 | *Ier3* | |  |  | -2.506 | 3.18E-02 |  |  |
| 9 | *EG330157* | |  |  | -2.368 | 7.38E-03 |  |  |
| 10 | *Trib3* | |  |  | -2.352 | 7.82E-02 |  |  |
| 11 | *Itpr2* | |  |  | -2.281 | 1.87E-02 |  |  |
| 12 | *Slc35c1* | |  |  | -2.147 | 2.19E-04 |  |  |
| 13 | *Bzrpl1* | |  |  | -2.079 | 2.71E-02 |  |  |
| 14 | *RP23-430I21.1* | |  |  | -1.942 | 3.32E-03 |  |  |
| 15 | *Slc46a3* | |  |  | -1.872 | 2.86E-02 |  |  |
| 16 | *Fzd5* | |  |  | -1.831 | 9.68E-02 |  |  |
| 17 | *Ikbkg* | |  |  | -1.714 | 1.13E-06 |  |  |
| 18 | *Lrg1* | |  |  | -1.680 | 1.92E-02 |  |  |
| 19 | *Pim1* | |  |  | -1.676 | 6.43E-03 |  |  |
| 20 | *Lsm10* | |  |  | -1.663 | 7.70E-03 |  |  |
| 21 | *Bola2* | |  |  | -1.646 | 3.00E-02 |  |  |
| 22 | *Trp53bp1* | |  |  | -1.637 | 8.50E-02 |  |  |
| 23 | *Ctsf* | |  |  | -1.624 | 7.75E-03 |  |  |
| 24 | *Ttyh3* | |  |  | -1.609 | 7.58E-02 |  |  |
| 25 | *Ms4a3* | |  |  | -1.600 | 5.52E-02 |  |  |
| 26 | *Fahd1* | |  |  | -1.599 | 2.12E-02 |  |  |
| 27 | *Rbms1* | |  |  | -1.582 | 4.31E-02 |  |  |
| 28 | *Prpf19* | |  |  | -1.575 | 8.01E-03 |  |  |
| 29 | *Wdr45l* | |  |  | -1.566 | 6.68E-02 |  |  |
| 30 | *Npm3* | |  |  | -1.556 | 3.33E-02 |  |  |
| 31 | *Bola1* | |  |  | -1.543 | 1.57E-02 |  |  |
| 32 | *Hdgf* | |  |  | -1.534 | 2.83E-04 |  |  |
| 33 | *S100a6* | |  |  | -1.532 | 1.63E-04 |  |  |
| 34 | *Ccdc86* | |  |  | -1.522 | 7.08E-02 |  |  |
| 35 | *Pink1* | |  |  | -1.509 | 1.07E-02 |  |  |
| 36 | *Rps2* | |  |  | -1.508 | 5.05E-04 |  |  |
| 37 | *Uxt* | |  |  | -1.505 | 1.29E-02 |  |  |
| 38 | *Fxc1* | |  |  | -1.502 | 3.71E-03 |  |  |
|  | | | | | | | | |
| **Sp day 4** | | |  | |  | | **Sp day 4** | |
|  | *Gene symbol* | |  |  |  |  | *fold change* | *p value* |
| 1 | *Serpina3k* | |  |  |  |  | -4.779 | 5.37E-02 |
| 2 | *Glipr2* | |  |  |  |  | -3.286 | 5.65E-02 |
| 3 | *Tcte3* | |  |  |  |  | -2.378 | 2.33E-02 |
| 4 | *2310039H08Rik* | |  |  |  |  | -2.325 | 2.28E-02 |
| 5 | *Bbs9* | |  |  |  |  | -2.125 | 2.59E-02 |
| 6 | *Tspan14* | |  |  |  |  | -2.074 | 3.07E-02 |
| 7 | *Arl6* | |  |  |  |  | -2.055 | 5.54E-03 |
| 8 | *Hsd17b7* | |  |  |  |  | -2.002 | 4.08E-02 |
| 9 | *Gdf3* | |  |  |  |  | -1.931 | 1.02E-03 |
| 10 | *Timp1* | |  |  |  |  | -1.854 | 1.58E-02 |
| 11 | *SPC22/23* | |  |  |  |  | -1.851 | 7.84E-03 |
| 12 | *Prmt5* | |  |  |  |  | -1.818 | 2.93E-03 |
| 13 | *Trm61* | |  |  |  |  | -1.765 | 5.67E-02 |
| 14 | *Vti1a* | |  |  |  |  | -1.734 | 4.85E-02 |
| 15 | *1810015A11Rik* | |  |  |  |  | -1.725 | 1.13E-02 |
| 16 | *Trmt6* | |  |  |  |  | -1.725 | 8.15E-03 |
| 17 | *Wdr74* | |  |  |  |  | -1.662 | 8.37E-04 |
| 18 | *Ccng2* | |  |  |  |  | -1.660 | 2.09E-02 |
| 19 | *Ebna1bp2* | |  |  |  |  | -1.656 | 4.01E-03 |
| 20 | *Slc19a1* | |  |  |  |  | -1.656 | 1.14E-02 |
| 21 | *Rpl38* | |  |  |  |  | -1.638 | 1.93E-02 |
| 22 | *Pold2* | |  |  |  |  | -1.628 | 6.47E-03 |
| 23 | *Gnl3* | |  |  |  |  | -1.626 | 2.65E-03 |
| 24 | *Exosc1* | |  |  |  |  | -1.621 | 2.62E-03 |
| 25 | *Bzw2* | |  |  |  |  | -1.616 | 1.28E-02 |
| 26 | *Wdr34* | |  |  |  |  | -1.611 | 3.29E-02 |
| 27 | *Gtf2f2* | |  |  |  |  | -1.604 | 4.59E-03 |
| 28 | *Srm* | |  |  |  |  | -1.602 | 1.98E-02 |
| 29 | *Rbm3* | |  |  |  |  | -1.598 | 4.81E-02 |
| 30 | *Chsy1* | |  |  |  |  | -1.596 | 1.18E-02 |
| 31 | *Aprt* | |  |  |  |  | -1.596 | 2.38E-02 |
| 32 | *BC039210* | |  |  |  |  | -1.590 | 6.87E-02 |
| 33 | *Mybbp1a* | |  |  |  |  | -1.583 | 4.43E-02 |
| 34 | *Tomm22* | |  |  |  |  | -1.580 | 6.00E-03 |
| 35 | *Lsm6* | |  |  |  |  | -1.575 | 3.79E-03 |
| 36 | *Tbrg4* | |  |  |  |  | -1.570 | 9.15E-03 |
| 37 | *Nufip2* | |  |  |  |  | -1.567 | 1.31E-02 |
| 38 | *Smpd3* | |  |  |  |  | -1.566 | 3.16E-03 |
| 39 | *Cirh1a* | |  |  |  |  | -1.564 | 6.14E-03 |
| 40 | *1110007M04Rik* | |  |  |  |  | -1.563 | 2.25E-02 |
| 41 | *Bcap29* | |  |  |  |  | -1.561 | 2.86E-02 |
| 42 | *Gtpbp4* | |  |  |  |  | -1.560 | 6.90E-03 |
| 43 | *2410002F23Rik* | |  |  |  |  | -1.559 | 5.79E-02 |
| 44 | *Mrto4* | |  |  |  |  | -1.558 | 1.59E-03 |
| 45 | *Mphosph6* | |  |  |  |  | -1.557 | 7.46E-03 |
| 46 | *Hist1h4h* | |  |  |  |  | -1.554 | 4.88E-03 |
| 47 | *Eif1a* | |  |  |  |  | -1.552 | 9.35E-02 |
| 48 | *Deadc1* | |  |  |  |  | -1.552 | 4.54E-02 |
| 49 | *Nupr1* | |  |  |  |  | -1.548 | 5.64E-02 |
| 50 | *Heatr1* | |  |  |  |  | -1.547 | 1.50E-02 |
| 51 | *Bex4* | |  |  |  |  | -1.539 | 6.09E-04 |
| 52 | *Sc4mol* | |  |  |  |  | -1.533 | 3.46E-02 |
| 53 | *Sdad1* | |  |  |  |  | -1.532 | 3.60E-02 |
| 54 | *Atad3a* | |  |  |  |  | -1.532 | 6.48E-03 |
| 55 | *Tmem5* | |  |  |  |  | -1.530 | 9.76E-02 |
| 56 | *Brp16* | |  |  |  |  | -1.529 | 3.89E-02 |
| 57 | *Thumpd1* | |  |  |  |  | -1.527 | 2.84E-02 |
| 58 | *Manbal* | |  |  |  |  | -1.524 | 9.44E-02 |
| 59 | *Snrpa* | |  |  |  |  | -1.522 | 3.30E-03 |
| 60 | *Cox10* | |  |  |  |  | -1.518 | 7.92E-02 |
| 61 | *Psat1* | |  |  |  |  | -1.516 | 4.25E-03 |
| 62 | *Gnl2* | |  |  |  |  | -1.511 | 2.66E-04 |
| 63 | *Hmgcs1* | |  |  |  |  | -1.505 | 3.92E-02 |
| 64 | *Hspa9* | |  |  |  |  | -1.505 | 2.54E-04 |
| 65 | *Uck2* | |  |  |  |  | -1.505 | 4.09E-04 |
| 66 | *Polr1e* | |  |  |  |  | -1.504 | 1.21E-02 |
| 67 | *Gtf2h1* | |  |  |  |  | -1.503 | 1.78E-03 |
| 68 | *Mrps18b* | |  |  |  |  | -1.503 | 4.48E-04 |
| 69 | *Nip7* | |  |  |  |  | -1.502 | 1.02E-02 |
| 70 | *Lsg1* | |  |  |  |  | -1.501 | 9.94E-04 |
| 71 | *Tmem85* | |  |  |  |  | -1.501 | 1.59E-02 |
|  | | | | | | | | |
| **Genes downregulated ≥1.5 fold. P value ≤0.1** | | | | | | | | |
|  | | | | | | | | |
| **All samples** | | | **BM day 0** | | **BM day 4** | | **Sp day 4** | |
|  | *Gene symbol* | | *fold change* | *p value* | *fold change* | *p value* | *fold change* | *p value* |
| 1 | *St5* | | 2.97 | 6.91E-03 | 3.00 | 1.00E-04 | 2.08 | 1.36E-03 |
| 2 | *Gda* | | 2.30 | 2.72E-03 | 1.84 | 1.48E-03 | 1.74 | 6.07E-02 |
| 3 | *Epor* | | 1.99 | 8.76E-03 | 2.18 | 1.59E-05 | 2.04 | 3.93E-05 |
| 4 | *Smox* | | 1.99 | 3.65E-02 | 2.40 | 6.28E-04 | 1.94 | 1.01E-02 |
| 5 | *Naprt1* | | 1.85 | 1.84E-02 | 2.30 | 4.43E-05 | 2.06 | 8.51E-05 |
| 6 | *Rnf11* | | 1.77 | 1.50E-02 | 1.88 | 5.11E-03 | 1.86 | 6.37E-02 |
| 7 | *Snx15* | | 1.75 | 3.98E-02 | 1.55 | 1.35E-02 | 1.50 | 6.03E-02 |
| 8 | *Wdr61* | | 1.73 | 8.97E-02 | 1.99 | 1.40E-04 | 1.68 | 9.22E-04 |
| 9 | *Eif5* | | 1.70 | 2.93E-02 | 1.84 | 7.39E-05 | 1.59 | 8.72E-03 |
| 10 | *St3gal5* | | 1.69 | 4.46E-02 | 1.68 | 1.07E-03 | 1.70 | 6.41E-03 |
| 11 | *Fbxo9* | | 1.62 | 9.08E-02 | 1.67 | 2.30E-02 | 1.77 | 4.19E-02 |
| 12 | *Igtp* | | 1.60 | 9.03E-02 | 1.78 | 3.88E-03 | 1.74 | 1.88E-02 |
|  | | | | | | | | |
| **BM days 0 and 4** | | | **BM day 0** | | **BM day 4** | |  | |
|  | *Gene symbol* | | *fold change* | *p value* | *fold change* | *p value* |  |  |
| 1 | *Wdr40a* | | 2.09 | 1.38E-02 | 1.99 | 1.24E-02 |  |  |
| 2 | *Lmo2* | | 1.96 | 6.50E-03 | 1.61 | 2.81E-03 |  |  |
| 3 | *Casp4* | | 1.89 | 1.00E-03 | 1.80 | 1.69E-02 |  |  |
| 4 | *Clec4d* | | 1.87 | 1.79E-03 | 1.88 | 2.32E-02 |  |  |
| 5 | *Slc38a5* | | 1.82 | 3.68E-02 | 1.53 | 1.58E-03 |  |  |
| 6 | *Slc14a1* | | 1.80 | 5.61E-02 | 1.87 | 4.27E-03 |  |  |
| 7 | *Csf3r* | | 1.78 | 6.49E-03 | 1.53 | 6.98E-02 |  |  |
| 8 | *BC013712* | | 1.76 | 7.77E-04 | 1.55 | 4.73E-02 |  |  |
| 9 | *Aqp9* | | 1.71 | 2.46E-02 | 1.92 | 3.73E-05 |  |  |
| 10 | *Pigq* | | 1.68 | 5.76E-02 | 1.50 | 6.58E-03 |  |  |
| 11 | *Slc6a4* | | 1.57 | 5.49E-02 | 1.54 | 9.52E-02 |  |  |
| 12 | *Ptpro* | | 1.56 | 2.88E-02 | 1.52 | 4.34E-02 |  |  |
| 13 | *Col5a1* | | 1.55 | 9.90E-02 | 1.58 | 3.84E-03 |  |  |
|  | | | | | | | | |
| **BM and Sp day 4** | | |  | | **BM day 4** | | **Sp day 4** | |
|  | *Gene symbol* | |  |  | *fold change* | *p value* | *fold change* | *p value* |
| 1 | *Sh3yl1* | |  |  | 2.93 | 9.21E-05 | 2.70 | 1.29E-04 |
| 2 | *Fgfr1op2* | |  |  | 2.83 | 5.44E-03 | 2.47 | 1.50E-02 |
| 3 | *5730469M10Rik* | |  |  | 2.61 | 1.90E-05 | 2.19 | 3.60E-04 |
| 4 | *AB182283* | |  |  | 2.45 | 9.58E-02 | 2.34 | 2.17E-02 |
| 5 | *Xpo7* | |  |  | 2.35 | 2.75E-05 | 2.58 | 5.89E-04 |
| 6 | *2810453I06Rik* | |  |  | 2.15 | 9.20E-03 | 2.12 | 5.63E-02 |
| 7 | *Pdzk1ip1* | |  |  | 2.15 | 1.43E-03 | 2.35 | 1.63E-03 |
| 8 | *Mboat5* | |  |  | 2.12 | 1.84E-04 | 1.92 | 1.85E-04 |
| 9 | *Ppp2r1b* | |  |  | 2.09 | 3.76E-05 | 1.52 | 6.83E-04 |
| 10 | *Tmcc2* | |  |  | 2.02 | 1.45E-05 | 2.28 | 1.11E-03 |
| 11 | *2700097O09Rik* | |  |  | 2.02 | 8.87E-03 | 2.03 | 2.47E-05 |
| 12 | *2810417H13Rik* | |  |  | 2.02 | 7.92E-04 | 1.76 | 1.75E-04 |
| 13 | *Otub2* | |  |  | 1.95 | 1.80E-02 | 1.71 | 2.67E-02 |
| 14 | *Acbd4* | |  |  | 1.93 | 1.80E-02 | 2.00 | 4.67E-03 |
| 15 | *Stard10* | |  |  | 1.92 | 1.99E-03 | 1.67 | 1.83E-04 |
| 16 | *Tlcd1* | |  |  | 1.92 | 2.97E-03 | 1.75 | 2.42E-03 |
| 17 | *Map3k7ip3* | |  |  | 1.92 | 5.73E-02 | 1.81 | 7.16E-02 |
| 18 | *Prnp* | |  |  | 1.91 | 5.30E-02 | 1.73 | 6.64E-02 |
| 19 | *Serinc1* | |  |  | 1.80 | 5.88E-03 | 1.90 | 3.40E-03 |
| 20 | *4432416J03Rik* | |  |  | 1.87 | 4.05E-02 | 1.53 | 5.66E-02 |
| 21 | *1190007F08Rik* | |  |  | 1.87 | 4.05E-02 | 1.53 | 5.66E-02 |
| 22 | *Aspm* | |  |  | 1.86 | 4.22E-04 | 1.54 | 1.70E-03 |
| 23 | *Tmem68* | |  |  | 1.86 | 1.08E-02 | 1.57 | 3.80E-02 |
| 24 | *Golph3l* | |  |  | 1.85 | 8.53E-04 | 1.50 | 1.55E-03 |
| 25 | *Cd82* | |  |  | 1.84 | 8.93E-04 | 1.55 | 5.09E-03 |
| 26 | *Metap2* | |  |  | 1.83 | 1.50E-03 | 1.52 | 8.24E-04 |
| 27 | *Gadd45a* | |  |  | 1.83 | 2.88E-03 | 1.58 | 1.92E-02 |
| 28 | *Pnp* | |  |  | 1.82 | 2.18E-02 | 1.65 | 7.54E-03 |
| 29 | *Scoc* | |  |  | 1.82 | 5.50E-02 | 1.53 | 4.39E-02 |
| 30 | *Pcmtd2* | |  |  | 1.80 | 1.14E-04 | 1.73 | 4.78E-04 |
| 31 | *Prei4* | |  |  | 1.79 | 1.01E-03 | 2.05 | 3.24E-03 |
| 32 | *Gpbp1* | |  |  | 1.79 | 3.21E-03 | 1.56 | 2.57E-03 |
| 33 | *Als2cr2* | |  |  | 1.77 | 1.26E-03 | 1.56 | 2.59E-03 |
| 34 | *Ppp1cb* | |  |  | 1.75 | 2.05E-03 | 1.56 | 3.10E-02 |
| 35 | *Fech* | |  |  | 1.74 | 5.37E-03 | 1.53 | 2.20E-02 |
| 36 | *Kntc1* | |  |  | 1.72 | 7.50E-03 | 1.55 | 3.14E-03 |
| 37 | *Slc16a10* | |  |  | 1.69 | 6.14E-03 | 1.84 | 3.73E-03 |
| 38 | *Ccne2* | |  |  | 1.67 | 2.77E-03 | 1.55 | 1.11E-04 |
| 39 | *Trak2* | |  |  | 1.66 | 2.52E-04 | 1.52 | 5.67E-03 |
| 40 | *Stxbp5l* | |  |  | 1.66 | 2.17E-02 | 1.82 | 6.50E-03 |
| 41 | *Ibtk* | |  |  | 1.64 | 3.63E-03 | 1.67 | 3.52E-03 |
| 42 | *C79407* | |  |  | 1.64 | 2.05E-03 | 1.60 | 7.75E-04 |
| 43 | *Rb1* | |  |  | 1.64 | 4.07E-05 | 1.56 | 2.37E-04 |
| 44 | *Atad2* | |  |  | 1.64 | 4.05E-03 | 1.56 | 4.20E-04 |
| 45 | *Ppap2a* | |  |  | 1.63 | 3.75E-03 | 1.57 | 6.06E-05 |
| 46 | *Emr1* | |  |  | 1.63 | 7.54E-02 | 1.51 | 8.88E-02 |
| 47 | *Osbpl8* | |  |  | 1.62 | 1.41E-04 | 1.88 | 1.35E-05 |
| 48 | *Cep164* | |  |  | 1.62 | 5.51E-04 | 1.57 | 3.42E-03 |
| 49 | *Cd24a* | |  |  | 1.56 | 9.95E-03 | 1.61 | 5.03E-02 |
| 50 | *Iigp2* | |  |  | 1.61 | 1.27E-02 | 1.59 | 3.67E-02 |
| 51 | *Tbcel* | |  |  | 1.58 | 7.28E-04 | 1.81 | 5.02E-03 |
| 52 | *Dnajb10* | |  |  | 1.58 | 1.58E-02 | 1.57 | 4.90E-02 |
| 53 | *Gse1* | |  |  | 1.58 | 2.68E-03 | 1.53 | 3.69E-02 |
| 54 | *Fancd2* | |  |  | 1.57 | 5.66E-03 | 1.52 | 5.44E-04 |
| 55 | *Ube2l6* | |  |  | 1.54 | 6.77E-02 | 1.52 | 8.84E-02 |
| 56 | *Sec14l2* | |  |  | 1.53 | 7.09E-02 | 1.54 | 7.29E-03 |
| 57 | *Capn1* | |  |  | 1.51 | 1.36E-02 | 1.57 | 1.84E-02 |
|  | | | | | | | | |
| **BM day 0** | | | **BM day 0** | |  | |  | |
|  | | *Gene symbol* | *fold change* | *p value* |  |  |  |  |
| 1 | | *Trp53inp1* | 5.71 | 6.21E-03 |  |  |  |  |
| 2 | | *Pnpo* | 3.10 | 3.40E-02 |  |  |  |  |
| 3 | | *Il8rb* | 2.63 | 8.07E-04 |  |  |  |  |
| 4 | | *Paqr9* | 2.33 | 1.65E-02 |  |  |  |  |
| 5 | | *Asprv1* | 2.17 | 8.59E-02 |  |  |  |  |
| 6 | | *Plxdc2* | 2.17 | 3.02E-02 |  |  |  |  |
| 7 | | *Ltf* | 2.13 | 5.96E-03 |  |  |  |  |
| 8 | | *Dapk2* | 2.10 | 3.17E-02 |  |  |  |  |
| 9 | | *Lrg1* | 2.08 | 1.01E-03 |  |  |  |  |
| 10 | | *NA* | 2.03 | 8.16E-02 |  |  |  |  |
| 11 | | *Slpi* | 2.03 | 9.39E-05 |  |  |  |  |
| 12 | | *Ankrd22* | 2.00 | 2.79E-03 |  |  |  |  |
| 13 | | *Pira3* | 1.97 | 6.41E-03 |  |  |  |  |
| 14 | | *Bcl2l1* | 1.96 | 3.45E-02 |  |  |  |  |
| 15 | | *Dfna5* | 1.94 | 4.50E-03 |  |  |  |  |
| 16 | | *Hk3* | 1.91 | 4.27E-03 |  |  |  |  |
| 17 | | *Mmp9* | 1.90 | 1.50E-03 |  |  |  |  |
| 18 | | *F730035M05Rik* | 1.89 | 1.66E-03 |  |  |  |  |
| 19 | | *Fcgr3a* | 1.89 | 4.65E-03 |  |  |  |  |
| 20 | | *Mrgpra2* | 1.89 | 6.85E-03 |  |  |  |  |
| 21 | | *Agpat2* | 1.88 | 5.32E-04 |  |  |  |  |
| 22 | | *Clec4b1* | 1.86 | 1.50E-02 |  |  |  |  |
| 23 | | *Tnfsf13b* | 1.85 | 1.27E-02 |  |  |  |  |
| 24 | | *Tlr13* | 1.85 | 8.91E-04 |  |  |  |  |
| 25 | | *Myo1f* | 1.84 | 3.03E-03 |  |  |  |  |
| 26 | | *Trem3* | 1.84 | 1.02E-03 |  |  |  |  |
| 27 | | *Mtus1* | 1.83 | 6.86E-04 |  |  |  |  |
| 28 | | *Clec5a* | 1.82 | 2.47E-03 |  |  |  |  |
| 29 | | *Mrgpra7* | 1.82 | 6.89E-03 |  |  |  |  |
| 30 | | *Emilin2* | 1.81 | 3.73E-03 |  |  |  |  |
| 31 | | *Fes* | 1.81 | 8.87E-03 |  |  |  |  |
| 32 | | *Gpr84* | 1.80 | 8.25E-03 |  |  |  |  |
| 33 | | *Ms4a3* | 1.80 | 6.60E-03 |  |  |  |  |
| 34 | | *Cebpe* | 1.79 | 1.84E-02 |  |  |  |  |
| 35 | | *Aldh3b1* | 1.79 | 5.11E-03 |  |  |  |  |
| 36 | | *Cd33* | 1.79 | 9.06E-04 |  |  |  |  |
| 37 | | *Il28ra* | 1.79 | 6.10E-03 |  |  |  |  |
| 38 | | *1100001G20Rik* | 1.79 | 1.98E-04 |  |  |  |  |
| 39 | | *Ncf1* | 1.79 | 1.07E-02 |  |  |  |  |
| 40 | | *F13a1* | 1.78 | 8.64E-03 |  |  |  |  |
| 41 | | *Sema4a* | 1.78 | 4.45E-03 |  |  |  |  |
| 42 | | *Anxa3* | 1.77 | 5.24E-03 |  |  |  |  |
| 43 | | *Sirpb1* | 1.77 | 3.02E-02 |  |  |  |  |
| 44 | | *Mmp28* | 1.75 | 5.99E-03 |  |  |  |  |
| 45 | | *4930598A11Rik* | 1.75 | 5.67E-02 |  |  |  |  |
| 46 | | *Retnla* | 1.75 | 7.73E-04 |  |  |  |  |
| 47 | | *Pira4* | 1.75 | 1.27E-02 |  |  |  |  |
| 48 | | *Nlrx1* | 1.74 | 2.21E-03 |  |  |  |  |
| 49 | | *sept8.* | 1.74 | 1.22E-02 |  |  |  |  |
| 50 | | *Slc25a45* | 1.74 | 5.18E-02 |  |  |  |  |
| 51 | | *Arl11* | 1.75 | 5.90E-03 |  |  |  |  |
| 52 | | *Tm6sf1* | 1.75 | 2.05E-02 |  |  |  |  |
| 53 | | *Kcnk6* | 1.74 | 6.98E-02 |  |  |  |  |
| 54 | | *Cldn15* | 1.72 | 1.11E-02 |  |  |  |  |
| 55 | | *2810470J21Rik* | 1.72 | 3.69E-03 |  |  |  |  |
| 56 | | *Ffar2* | 1.72 | 5.67E-02 |  |  |  |  |
| 57 | | *Lilrb3* | 1.72 | 1.59E-02 |  |  |  |  |
| 58 | | *Upp1* | 1.71 | 2.21E-03 |  |  |  |  |
| 59 | | *Csf2ra* | 1.71 | 1.09E-02 |  |  |  |  |
| 60 | | *Ccl9* | 1.70 | 7.76E-04 |  |  |  |  |
| 61 | | *cd82* | 1.70 | 5.02E-02 |  |  |  |  |
| 62 | | *Cyp4f18* | 1.70 | 5.87E-03 |  |  |  |  |
| 63 | | *Dock5* | 1.69 | 8.66E-03 |  |  |  |  |
| 64 | | *Slfn1* | 1.69 | 4.46E-02 |  |  |  |  |
| 65 | | *Ctsg* | 1.69 | 1.27E-02 |  |  |  |  |
| 66 | | *Lyzs* | 1.68 | 1.14E-02 |  |  |  |  |
| 67 | | *Pira11* | 1.68 | 1.18E-02 |  |  |  |  |
| 68 | | *LOC100034251* | 1.68 | 5.85E-02 |  |  |  |  |
| 69 | | *Inhba* | 1.68 | 2.38E-03 |  |  |  |  |
| 70 | | *Lst1* | 1.67 | 3.24E-02 |  |  |  |  |
| 71 | | *Anxa1* | 1.67 | 1.34E-02 |  |  |  |  |
| 72 | | *Nqo2* | 1.67 | 1.21E-02 |  |  |  |  |
| 73 | | *F2rl3* | 1.67 | 5.31E-03 |  |  |  |  |
| 74 | | *Apob48r* | 1.66 | 1.96E-02 |  |  |  |  |
| 75 | | *Sell* | 1.66 | 9.71E-02 |  |  |  |  |
| 76 | | *5830415F09Rik* | 1.65 | 1.03E-02 |  |  |  |  |
| 77 | | *Itgb2l* | 1.65 | 2.18E-02 |  |  |  |  |
| 78 | | *Ncf4* | 1.64 | 2.12E-02 |  |  |  |  |
| 79 | | *Tmem216* | 1.64 | 1.20E-02 |  |  |  |  |
| 80 | | *Chi3l1* | 1.64 | 8.68E-03 |  |  |  |  |
| 81 | | *Mgst1* | 1.64 | 3.88E-03 |  |  |  |  |
| 82 | | *Zmpste24* | 1.64 | 4.40E-03 |  |  |  |  |
| 83 | | *Ccdc125* | 1.63 | 3.25E-03 |  |  |  |  |
| 84 | | *Il18rap* | 1.63 | 3.08E-03 |  |  |  |  |
| 85 | | *Dgkg* | 1.63 | 6.78E-03 |  |  |  |  |
| 86 | | *Nfe2l2* | 1.63 | 2.06E-02 |  |  |  |  |
| 87 | | *Rdh12* | 1.62 | 1.33E-03 |  |  |  |  |
| 88 | | *Sirpa* | 1.62 | 4.49E-02 |  |  |  |  |
| 89 | | *Tyrobp* | 1.62 | 1.42E-03 |  |  |  |  |
| 90 | | *AI504432* | 1.61 | 4.04E-03 |  |  |  |  |
| 91 | | *Ddef1* | 1.61 | 2.63E-03 |  |  |  |  |
| 92 | | *Hsd11b1* | 1.61 | 3.91E-03 |  |  |  |  |
| 93 | | *Mocos* | 1.61 | 4.61E-03 |  |  |  |  |
| 94 | | *Gp1ba* | 1.61 | 8.10E-04 |  |  |  |  |
| 95 | | *Gca* | 1.61 | 1.26E-03 |  |  |  |  |
| 96 | | *Gbl* | 1.61 | 1.10E-02 |  |  |  |  |
| 97 | | *F10* | 1.61 | 1.79E-02 |  |  |  |  |
| 98 | | *Tspan32* | 1.60 | 7.02E-02 |  |  |  |  |
| 99 | | *Rnf123* | 1.60 | 2.92E-03 |  |  |  |  |
| 100 | | *Klra17* | 1.60 | 5.98E-02 |  |  |  |  |
| 101 | | *Amica1* | 1.60 | 1.01E-02 |  |  |  |  |
| 102 | | *Pik3cg* | 1.60 | 1.02E-02 |  |  |  |  |
| 103 | | *Ptplad2* | 1.60 | 5.07E-03 |  |  |  |  |
| 104 | | *Aytl1* | 1.59 | 2.29E-03 |  |  |  |  |
| 105 | | *Scrg1* | 1.59 | 1.00E-02 |  |  |  |  |
| 106 | | *Cd84* | 1.59 | 2.75E-02 |  |  |  |  |
| 107 | | *Pglyrp1* | 1.59 | 1.41E-02 |  |  |  |  |
| 108 | | *Eea1* | 1.59 | 8.91E-02 |  |  |  |  |
| 109 | | *Rhou* | 1.59 | 5.44E-03 |  |  |  |  |
| 110 | | *Orm2* | 1.58 | 1.14E-02 |  |  |  |  |
| 111 | | *Gfi1b* | 1.58 | 6.82E-02 |  |  |  |  |
| 112 | | *dhrs7* | 1.58 | 9.41E-04 |  |  |  |  |
| 113 | | *Plcl2* | 1.58 | 3.35E-02 |  |  |  |  |
| 114 | | *Mrm1* | 1.58 | 1.46E-02 |  |  |  |  |
| 115 | | *Aatk* | 1.58 | 4.54E-02 |  |  |  |  |
| 116 | | *Srd5a3* | 1.58 | 2.76E-02 |  |  |  |  |
| 117 | | *Sc4mol* | 1.57 | 4.59E-02 |  |  |  |  |
| 118 | | *Rnpep* | 1.57 | 9.71E-03 |  |  |  |  |
| 119 | | *Cd68* | 1.57 | 3.80E-03 |  |  |  |  |
| 120 | | *Centd3* | 1.57 | 7.93E-03 |  |  |  |  |
| 121 | | *Rab32* | 1.57 | 4.11E-02 |  |  |  |  |
| 122 | | *Mppe1* | 1.57 | 2.57E-02 |  |  |  |  |
| 123 | | *Skap2* | 1.56 | 4.84E-03 |  |  |  |  |
| 124 | | *Fcnb* | 1.56 | 1.43E-02 |  |  |  |  |
| 125 | | *Psme3* | 1.55 | 4.46E-02 |  |  |  |  |
| 126 | | *Sidt2* | 1.55 | 9.84E-03 |  |  |  |  |
| 127 | | *Mgst2* | 1.55 | 6.95E-02 |  |  |  |  |
| 128 | | *Xdh* | 1.55 | 3.82E-03 |  |  |  |  |
| 129 | | *Nadk* | 1.55 | 5.88E-03 |  |  |  |  |
| 130 | | *B430306N03Rik* | 1.55 | 1.83E-02 |  |  |  |  |
| 131 | | *Tacstd2* | 1.55 | 6.35E-03 |  |  |  |  |
| 132 | | *Paqr7* | 1.55 | 2.99E-03 |  |  |  |  |
| 133 | | *Fntb* | 1.54 | 3.27E-02 |  |  |  |  |
| 134 | | *Gng2* | 1.54 | 4.00E-02 |  |  |  |  |
| 135 | | *Slc15a3* | 1.54 | 2.34E-02 |  |  |  |  |
| 136 | | *Dhrs7* | 1.54 | 2.61E-03 |  |  |  |  |
| 137 | | *Nfam1* | 1.54 | 4.51E-03 |  |  |  |  |
| 138 | | *Rac2* | 1.53 | 4.59E-02 |  |  |  |  |
| 139 | | *Tgfbi* | 1.53 | 1.33E-02 |  |  |  |  |
| 140 | | *Ceacam1* | 1.53 | 1.75E-02 |  |  |  |  |
| 141 | | *Garnl4* | 1.53 | 1.24E-02 |  |  |  |  |
| 142 | | *Idh1* | 1.53 | 1.15E-02 |  |  |  |  |
| 143 | | *Fgr* | 1.53 | 2.53E-02 |  |  |  |  |
| 144 | | *Ifitm6* | 1.52 | 1.01E-02 |  |  |  |  |
| 145 | | *Mgl1* | 1.52 | 2.41E-02 |  |  |  |  |
| 146 | | *Abhd5* | 1.51 | 8.47E-03 |  |  |  |  |
| 147 | | *Il1b* | 1.51 | 2.36E-02 |  |  |  |  |
| 148 | | *Serpinb1a* | 1.51 | 6.61E-03 |  |  |  |  |
| 149 | | *Sphk1* | 1.51 | 9.70E-02 |  |  |  |  |
| 150 | | *C3* | 1.51 | 7.94E-03 |  |  |  |  |
| 151 | | *G6pdx* | 1.51 | 1.10E-03 |  |  |  |  |
| 152 | | *Ahnak* | 1.50 | 1.57E-02 |  |  |  |  |
| 153 | | *Abcc3* | 1.50 | 6.62E-02 |  |  |  |  |
| 154 | | *Rffl* | 1.50 | 2.04E-02 |  |  |  |  |
|  | | | | | | | | |
| **BM day 4** | | |  | | **BM day 4** | |  | |
|  | | *Gene symbol* |  |  | *fold change* | *p value* |  |  |
| 1 | | *Slc15a2* |  |  | 3.51 | 4.61E-02 |  |  |
| 2 | | *Prg3* |  |  | 3.44 | 3.10E-02 |  |  |
| 3 | | *Isg15* |  |  | 2.58 | 4.25E-02 |  |  |
| 4 | | *Fn3k* |  |  | 2.50 | 9.76E-04 |  |  |
| 5 | | *Epx* |  |  | 2.48 | 6.19E-02 |  |  |
| 6 | | *Trib2* |  |  | 2.22 | 5.56E-02 |  |  |
| 7 | | *Cbfa2t3h* |  |  | 2.19 | 2.88E-03 |  |  |
| 8 | | *Gabarapl2* |  |  | 2.09 | 1.50E-03 |  |  |
| 9 | | *Ghitm* |  |  | 2.06 | 8.16E-03 |  |  |
| 10 | | *Ppbp* |  |  | 2.04 | 1.35E-03 |  |  |
| 11 | | *BC021614* |  |  | 2.00 | 1.90E-04 |  |  |
| 12 | | *Ube2b* |  |  | 1.95 | 8.23E-03 |  |  |
| 13 | | *Uros* |  |  | 1.95 | 1.80E-02 |  |  |
| 14 | | *Wdhd1* |  |  | 1.89 | 1.84E-04 |  |  |
| 15 | | *Kif18a* |  |  | 1.84 | 3.11E-03 |  |  |
| 16 | | *Ubadc1* |  |  | 1.83 | 4.08E-03 |  |  |
| 17 | | *Klhl12* |  |  | 1.83 | 5.51E-02 |  |  |
| 18 | | *Tspan8* |  |  | 1.81 | 4.06E-03 |  |  |
| 19 | | *Zfp367* |  |  | 1.81 | 4.25E-02 |  |  |
| 20 | | *Mospd1* |  |  | 1.81 | 1.94E-03 |  |  |
| 21 | | *1700027F06Rik* |  |  | 1.81 | 1.64E-03 |  |  |
| 22 | | *Mcm6* |  |  | 1.80 | 1.60E-02 |  |  |
| 23 | | *Plek* |  |  | 1.80 | 1.13E-04 |  |  |
| 24 | | *Cul4a* |  |  | 1.79 | 2.42E-04 |  |  |
| 25 | | *Pcna* |  |  | 1.79 | 3.28E-03 |  |  |
| 26 | | *Plk4* |  |  | 1.78 | 1.41E-03 |  |  |
| 27 | | *Prpf38b* |  |  | 1.76 | 3.59E-02 |  |  |
| 28 | | *Butr1* |  |  | 1.75 | 5.24E-04 |  |  |
| 29 | | *Csda* |  |  | 1.75 | 7.01E-04 |  |  |
| 30 | | *Aytl2* |  |  | 1.72 | 6.38E-05 |  |  |
| 31 | | *Isca1* |  |  | 1.72 | 3.23E-02 |  |  |
| 32 | | *Aqp1* |  |  | 1.71 | 1.49E-03 |  |  |
| 33 | | *Arg2* |  |  | 1.69 | 3.78E-03 |  |  |
| 34 | | *Prg2* |  |  | 1.68 | 1.14E-02 |  |  |
| 35 | | *Tfdp2* |  |  | 1.68 | 4.30E-02 |  |  |
| 36 | | *Memo1* |  |  | 1.67 | 2.63E-03 |  |  |
| 37 | | *Stk17b* |  |  | 1.67 | 8.57E-02 |  |  |
| 38 | | *SNX1* |  |  | 1.67 | 1.19E-02 |  |  |
| 39 | | *Ptdss2* |  |  | 1.66 | 3.97E-03 |  |  |
| 40 | | *Sepp1* |  |  | 1.66 | 8.05E-02 |  |  |
| 41 | | *Prss34* |  |  | 1.64 | 5.17E-02 |  |  |
| 42 | | *Epb4.1* |  |  | 1.64 | 1.38E-02 |  |  |
| 43 | | *Snx22* |  |  | 1.63 | 1.99E-03 |  |  |
| 44 | | *Acsl1* |  |  | 1.63 | 2.40E-02 |  |  |
| 45 | | *A930008G19Rik* |  |  | 1.62 | 6.24E-04 |  |  |
| 46 | | *Tax1bp1* |  |  | 1.62 | 3.30E-04 |  |  |
| 47 | | *Hnrpa2b1* |  |  | 1.61 | 2.93E-02 |  |  |
| 48 | | *Cdk5rap1* |  |  | 1.61 | 3.89E-02 |  |  |
| 49 | | *Polq* |  |  | 1.61 | 2.14E-02 |  |  |
| 50 | | *Mier1* |  |  | 1.61 | 1.26E-03 |  |  |
| 51 | | *Zdhhc14* |  |  | 1.60 | 9.42E-04 |  |  |
| 52 | | *Yod1* |  |  | 1.60 | 2.51E-02 |  |  |
| 53 | | *Akap7* |  |  | 1.60 | 2.09E-02 |  |  |
| 54 | | *Mpp1* |  |  | 1.60 | 2.65E-02 |  |  |
| 55 | | *Cyb5* |  |  | 1.60 | 1.57E-02 |  |  |
| 56 | | *Optn* |  |  | 1.59 | 5.54E-03 |  |  |
| 57 | | *Sass6* |  |  | 1.59 | 1.41E-02 |  |  |
| 58 | | *Pcmt1* |  |  | 1.59 | 4.27E-03 |  |  |
| 59 | | *Itsn2* |  |  | 1.59 | 9.19E-02 |  |  |
| 60 | | *Stx2* |  |  | 1.59 | 6.62E-03 |  |  |
| 61 | | *Otud5* |  |  | 1.59 | 2.23E-04 |  |  |
| 62 | | *Zfyve21* |  |  | 1.59 | 2.03E-03 |  |  |
| 63 | | *Cdr2* |  |  | 1.58 | 6.54E-02 |  |  |
| 64 | | *Elf1* |  |  | 1.58 | 5.29E-02 |  |  |
| 65 | | *A130092J06Rik* |  |  | 1.58 | 2.08E-02 |  |  |
| 66 | | *Nat2* |  |  | 1.58 | 1.51E-02 |  |  |
| 67 | | *Gcnt1* |  |  | 1.58 | 9.56E-04 |  |  |
| 68 | | *2410017P07Rik* |  |  | 1.57 | 5.02E-02 |  |  |
| 69 | | *Cdc6* |  |  | 1.56 | 2.96E-03 |  |  |
| 70 | | *Tcea1* |  |  | 1.56 | 4.04E-04 |  |  |
| 71 | | *Usp15* |  |  | 1.56 | 3.72E-02 |  |  |
| 72 | | *Cmas* |  |  | 1.55 | 3.62E-02 |  |  |
| 73 | | *Prim2* |  |  | 1.55 | 5.55E-04 |  |  |
| 74 | | *Adam17* |  |  | 1.55 | 1.96E-02 |  |  |
| 75 | | *Zfp68* |  |  | 1.55 | 7.55E-02 |  |  |
| 76 | | *2610204L23Rik* |  |  | 1.55 | 3.23E-02 |  |  |
| 77 | | *Dnase1l1* |  |  | 1.55 | 7.25E-03 |  |  |
| 78 | | *Fignl1* |  |  | 1.54 | 2.10E-02 |  |  |
| 79 | | *Paip2* |  |  | 1.54 | 2.09E-04 |  |  |
| 80 | | *Isg20* |  |  | 1.54 | 3.96E-02 |  |  |
| 81 | | *Eef2k* |  |  | 1.54 | 2.24E-04 |  |  |
| 82 | | *Gch1* |  |  | 1.54 | 4.12E-02 |  |  |
| 83 | | *Fbxo3* |  |  | 1.53 | 1.36E-02 |  |  |
| 84 | | *Anln* |  |  | 1.53 | 5.45E-03 |  |  |
| 85 | | *Jak2* |  |  | 1.53 | 6.19E-02 |  |  |
| 86 | | *Rfwd3* |  |  | 1.53 | 2.54E-04 |  |  |
| 87 | | *Tnfaip2* |  |  | 1.53 | 8.86E-02 |  |  |
| 88 | | *Zfp39* |  |  | 1.53 | 2.69E-03 |  |  |
| 89 | | *Matr3* |  |  | 1.52 | 2.77E-02 |  |  |
| 90 | | *1110038D17Rik* |  |  | 1.52 | 6.28E-05 |  |  |
| 91 | | *Cse1l* |  |  | 1.52 | 4.82E-02 |  |  |
| 92 | | *Setd8* |  |  | 1.52 | 6.27E-05 |  |  |
| 93 | | *Zfp672* |  |  | 1.52 | 3.02E-02 |  |  |
| 94 | | *Cxcl4* |  |  | 1.52 | 4.99E-02 |  |  |
| 95 | | *Slc25a3* |  |  | 1.52 | 7.74E-02 |  |  |
| 96 | | *Zranb3* |  |  | 1.52 | 8.87E-03 |  |  |
| 97 | | *Nav1* |  |  | 1.52 | 4.95E-02 |  |  |
| 98 | | *Rsu1* |  |  | 1.52 | 1.82E-02 |  |  |
| 99 | | *Fusip1* |  |  | 1.51 | 1.01E-02 |  |  |
| 100 | | *Smchd1* |  |  | 1.51 | 3.96E-03 |  |  |
| 101 | | *Smc6* |  |  | 1.51 | 8.49E-04 |  |  |
| 102 | | *Clk4* |  |  | 1.51 | 4.59E-02 |  |  |
| 103 | | *Ube2f* |  |  | 1.51 | 7.41E-03 |  |  |
| 104 | | *Ypel4* |  |  | 1.51 | 9.81E-03 |  |  |
| 105 | | *Wnk1* |  |  | 1.51 | 4.64E-04 |  |  |
| 106 | | *Ube2t* |  |  | 1.50 | 3.49E-02 |  |  |
| 107 | | *Dennd2c* |  |  | 1.50 | 1.34E-03 |  |  |
| 108 | | *Ckap2l* |  |  | 1.50 | 3.20E-03 |  |  |
| 109 | | *Tmem14c* |  |  | 1.50 | 1.26E-02 |  |  |
| 110 | | *Golph2* |  |  | 1.50 | 5.60E-03 |  |  |
|  | | | | | | | | |
| **Sp day 4** | | |  | |  | | **Sp day 4** | |
|  | *Gene symbol* | |  |  |  |  | *fold change* | *p value* |
| 1 | *Fn3k* | |  |  |  |  | 2.04 | 4.63E-03 |
| 2 | *Ube2b* | |  |  |  |  | 1.99 | 1.97E-02 |
| 3 | *Ypel3* | |  |  |  |  | 1.80 | 2.35E-02 |
| 4 | *D930015E06Rik* | |  |  |  |  | 1.78 | 3.03E-04 |
| 5 | *Ypel4* | |  |  |  |  | 1.77 | 6.80E-03 |
| 6 | *Mrc1* | |  |  |  |  | 1.76 | 1.39E-02 |
| 7 | *Ghtim* | |  |  |  |  | 1.72 | 2.36E-02 |
| 8 | *csdA* | |  |  |  |  | 1.71 | 2.98E-04 |
| 9 | *Vcam1* | |  |  |  |  | 1.70 | 9.70E-03 |
| 10 | *Sass6* | |  |  |  |  | 1.67 | 2.87E-03 |
| 11 | *Aldh1a1* | |  |  |  |  | 1.67 | 4.23E-02 |
| 12 | *Ccdc80* | |  |  |  |  | 1.65 | 6.23E-03 |
| 13 | *Pik3r1* | |  |  |  |  | 1.64 | 5.26E-03 |
| 14 | *Supt3h* | |  |  |  |  | 1.63 | 2.53E-02 |
| 15 | *Abhd4* | |  |  |  |  | 1.62 | 1.11E-02 |
| 16 | *Isca1* | |  |  |  |  | 1.61 | 3.51E-02 |
| 17 | *bc021614* | |  |  |  |  | 1.61 | 3.72E-04 |
| 18 | *Hist4h4* | |  |  |  |  | 1.60 | 7.19E-02 |
| 19 | *Abcb10* | |  |  |  |  | 1.60 | 1.69E-03 |
| 20 | *Tmsb4x* | |  |  |  |  | 1.59 | 8.91E-03 |
| 21 | *Senp7* | |  |  |  |  | 1.58 | 6.28E-02 |
| 22 | *Arhgap18* | |  |  |  |  | 1.58 | 1.23E-03 |
| 23 | *Zfand6* | |  |  |  |  | 1.58 | 3.75E-02 |
| 24 | *Klhl7* | |  |  |  |  | 1.57 | 1.51E-02 |
| 25 | *Mxi1* | |  |  |  |  | 1.57 | 2.27E-02 |
| 26 | *Cav1* | |  |  |  |  | 1.56 | 4.29E-03 |
| 27 | *Crat* | |  |  |  |  | 1.54 | 2.75E-03 |
| 28 | *Prkra* | |  |  |  |  | 1.54 | 1.76E-02 |
| 29 | *Hbp1* | |  |  |  |  | 1.53 | 1.29E-02 |
| 30 | *Dpp7* | |  |  |  |  | 1.53 | 3.13E-02 |
| 31 | *March2* | |  |  |  |  | 1.52 | 8.02E-02 |
| 32 | *Wnk1* | |  |  |  |  | 1.51 | 5.36E-03 |
| 33 | *Pip5k2a* | |  |  |  |  | 1.50 | 2.15E-02 |
